# Enhanced metastasis risk prediction in cutaneous squamous cell carcinoma using deep learning and computational histopathology

**DOI:** 10.1038/s41698-025-01065-7

**Published:** 2025-09-02

**Authors:** Emilia Peleva, Yue Chen, Bernhard Finke, Hasan Rizvi, Eugene Healy, Chester Lai, Paul Craig, William Rickaby, Christina Schoenherr, Craig Nourse, Charlotte Proby, Gareth J. Inman, Irene M. Leigh, Catherine A. Harwood, Jun Wang

**Affiliations:** 1 Centre for Cancer Evolution, Barts Cancer Institute, Queen Mary University of London, London, UK; 2 Dermatology, The Royal London Hospital, Barts Health NHS Trust, London, UK; 3 Pathology, The Royal London Hospital, Barts Health NHS Trust, London, UK; 4 Centre for Cell Biology and Cutaneous Research, Queen Mary University of London, London, UK; 5 Dermatopharmacology, Faculty of Medicine, University of Southampton, Southampton, UK; 6 Dermatology, University Hospital Southampton NHS Foundation Trust, Southampton, UK; 7 Department of Medicine and Therapeutics, Li Ka Shing Institute of Health Sciences, Faculty of Medicine, The Chinese University of Hong Kong, Hong Kong, China; 8 Cellular Pathology, Cheltenham General Hospital, Gloucestershire Hospitals NHS Foundation Trust, Cheltenham, UK; 9 Cellular Pathology, Southmead Hospital, North Bristol NHS Trust, Bristol, UK; 10 Cellular Pathology, University College London Hospitals NHS Foundation Trust, London, UK; 11 Cancer Research UK Scotland Institute, Glasgow, UK; 12 Botton-Champalimaud Pancreatic Cancer Centre, Lisboa, Portugal; 13 Division of Cancer Research, School of Medicine, University of Dundee, Dundee, UK; 14 School of Cancer Sciences, College of Medicine, Veterinary and Life Sciences, University of Glasgow, Glasgow, UK; 15 Barts Centre for Squamous Cancer, Faculty of Medicine and Dentistry, Queen Mary University of London, London, UK

**Keywords:** Squamous cell carcinoma, Translational research

## Abstract

Cutaneous squamous cell carcinoma (cSCC) is the most common skin cancer with metastatic potential and development of metastases carries a poor prognosis. To address the need for reliable risk stratification, we developed cSCCNet, a deep learning model using digital pathology of primary cSCC to predict metastatic risk. A retrospective cohort of 227 primary cSCC from four centres is used for model development. cSCCNet automatically selects the tumour area in standard histopathological slides and then stratifies primary cSCC into high- vs. low-risk categories, with heatmaps indicating most predictive tiles contributing to explainability. On a 20% hold-out testing cohort, cSCCNet achieves an area under the curve (AUC) of 0.95 and 95% accuracy in predicting risk of metastasis, outperforming gene expression-based tools and clinicopathologic classifications. Multivariate analysis including common clinicopathologic classifications confirms cSCCNet as an independent predictor for metastasis, implying it identifies predictive features beyond known clinicopathologic risk factors. Histopathological analysis including multiplex immunohistochemistry suggests that tumour differentiation, acantholysis, desmoplasia, and the spatial localisation of lymphocytes relative to tumour tissue may be important in predicting risk of developing metastasis. Although further validation including prospective evaluation is required, cSCCNet has potential as a reliable and accurate tool for metastatic risk prediction that could be easily integrated into existing histopathology workflows.

## Introduction

Cutaneous squamous cell carcinoma (cSCC) is the second most common skin cancer, after basal cell carcinoma (BCC), and presents a significant global public health challenge, with >300,000 new cases annually in Australia, 1 million in the United States and >50,000 in the United Kingdom (UK)^1,2^. Although most cSCC are curable by surgery and radiotherapy, absolute numbers are high and outcomes for metastatic cSCC (a risk rate of 2–5%) are poor^3,4^. In the UK, the three-year survival for metastatic cSCC was 29–46% in 2015^4^. cSCC incidence is increasing by approximately 5% per year, partly due to an aging population. If current trends continue, deaths from keratinocyte cancers (BCC and cSCC) are estimated to overtake deaths from melanoma in the UK, USA, northern Europe and Australia in the next 10–15 years^5–7^. Ideally, risk-based patient stratification would identify all patients with cSCC at high risk of developing metastasis who could benefit from more intensive management regimes, as well as all low-risk patients who may not need prolonged clinical surveillance after surgery. Current risk stratification strategies rely on clinicopathologic risk factors, but the commonly-used staging and/or classification criteria, including the closely aligned 8th edition American Joint Committee on Cancer staging manual (AJCC8) and 8th edition Union for International Cancer Control criteria (UICC8), as well as the Brigham and Women’s Hospital classification (BWH) are relatively poor at predicting metastasis, with positive predictive values (PPV) of 4.5–30%^8,9^. There are additional limitations to staging, including poor inter-rater reliability^10^. Two clinicopathological prognostic models have recently been developed by Rentroia-Pacheco et al. (2023)^11^ and Jambusaria-Pahlajani et al. (2025)^12^ that showed improved predictive performance for metastatic risk compared to staging systems. However, these models require further validation, and more benefits may be obtained by incorporating additional data types, for instance, molecular and more detailed histopathology data.

There is an increasing body of evidence focusing on the molecular landscape of primary cSCC, with the aim of identifying prognostic biomarkers and novel therapeutic targets. Examples include a 40-gene expression profile (GEP) signature developed by Castle Biosciences Inc. (Texas, USA)^13^ and our recently developed 20-GEP signature from a whole-transcriptome discovery effort to predict metastatic risk of primary cSCC^14^. In addition, proteomic analysis has identified proteins associated with the development of metastases from cSCC^15^. Although promising, these molecular prognostic signatures require evaluation and refinement in larger and diverse nationwide case-control cohorts before prospective clinical evaluation. There are additional limitations to a gene signature approach, as this method requires good RNA quality and quantity, which can be difficult to obtain from archival formalin-fixed paraffin-embedded (FFPE) samples. Likewise, challenges exist in relation to obtaining relevant proteins from FFPE samples. These approaches may also be time-consuming and expensive to incorporate into routine clinical practice. Therefore, robust, affordable, unbiased and easy-to-use prognostic tools for metastasis risk prediction would be a major step forward in guiding management strategies, to improve patient outcomes and to optimise use of healthcare resources.

Digital pathology, which involves scanning histopathological slides to produce whole slide images (WSI), is becoming increasingly accessible worldwide. Deep learning (DL) models trained on WSI can identify the presence of specific morphological features and gene mutations, and can even predict prognostic outcomes in a variety of cancer types^16–19^. Kulkarni et al.(2020)^20^ recently published a DL model trained on haematoxylin and eosin-stained (H&E) WSI of melanoma to predict the risk of visceral recurrence and death. In cSCC, the application of artificial intelligence (AI) on WSI to predict clinical outcomes is still relatively limited compared to melanoma, partly due to lack of large patient cohorts with well-defined outcomes and annotated images for model training. Few DL studies have addressed prediction of cSCC metastasis from WSI. Knuutila et al. (2022)^21^ developed a ResNet18-based model using a single-centre cohort; however, its performance was inferior to that of standard clinicopathologic classification systems. Moreover, this approach relied on manual region of interest (ROI) annotation by pathologists, a limitation addressed by our current study. More recently, Coudray et al. (2025)^22^ developed a model that predicts poor outcomes in cSCC based on the abundance of different ‘histomorphological phenotype clusters’ (HPCs), and Pisula et al.(2025)^23^ used a transformer-based architecture to predict cSCC progression. Notably, Pisula et al. demonstrate that models trained on multi-centre data outperform those trained on single-centre datasets, supporting the rationale for the multi-centre approach in the present study^23^.

In this study, leveraging our recently assembled multicentre UK cohort of 227 patients with primary cSCC with known metastasis outcomes and associated clinical archival tissue, we present the development and evaluation of cSCCNet, a two-step DL model for predicting metastatic risk from WSI of primary cSCC. In order to eliminate the need for time-consuming pathologist annotations, cSCCNet first selects the prognostically relevant area within a WSI and then predicts metastatic risk. We show that our histology AI model outperforms conventional clinicopathologic classifications and our recently developed 20-GEP molecular model, and is an independent predictor from histopathological classifications.

## Results

### Overview of cSCCNet

**Fig. 1:**
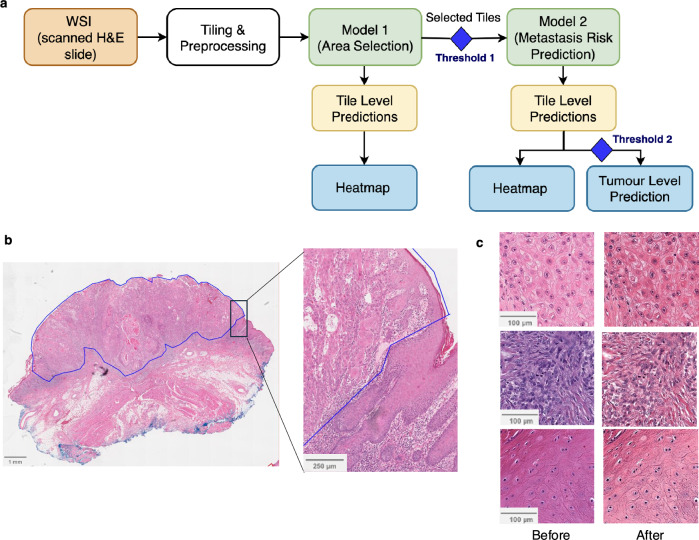
The development and overview of the cSCCNet model. **a** cSCCNet data pathway is shown, including input in orange, automated preprocessing steps in white, trained models in green, initial model outputs in yellow, thresholds as blue diamonds and user-facing predictions in blue. Threshold 1: scores ≥0.65. Threshold 2: median score >0.2 after excluding tiles with Model 2 scores of 0.3–0.7. **b** Whole slide image, with pathologist-annotated region of interest (ROI) delineated in blue. **c** Representative image tiles (*n* = 3) before and after colour normalisation.

cSCCNet consists of two models: Model 1 for ‘automated area selection’ and Model 2 for ‘prediction of metastatic risk’ (Fig. 1a). As WSI often contain artefacts and normal tissue that can potentially confound the prediction of tumour characteristics and behaviour, our Model 1 automatically selects ROI. ROI are defined as tumour, intratumoral inflammatory cells, and peri-tumoral stroma, as these have been shown to contribute to tumour progression^24^. Tiles within ROI are then extracted and used as input for Model 2 to predict metastatic risk for the sample of interest. Tile-level prediction is performed first to determine the predictive risk of each tile within a WSI. Informative tiles (i.e., confidently labelled high–risk or low-risk for metastasis by the model) are identified, and data is then amalgamated to generate a tumour-level prediction of metastasis (Fig. 1a).

### Development of Model 1 – automated area selection

To develop Model 1, WSI from all 227 cSCC from four centres (Supplementary Fig. 1a) were first annotated by an expert dermatopathologist (HR) to select the ROI, defined as all tumour regions, intratumoral inflammatory cells, and thin rim of peri-tumoral stroma (Fig. 1b). Non-overlapping image tiles of 512 × 512 pixels at 20x magnification were extracted, resulting in 167,814 ROI and 295,665 non-ROI tiles. Following colour normalisation (Fig. 1c) and the separation of the hold-out testing cohort, Model 1 was trained on the remaining 145,425 ROI and 237,069 non-ROI tiles from the training cohort (n = 187 cSCC). The split into training and testing cohorts is explained in Supplementary Fig. 1a. KerasTuner^25^ was used for systematic comparison of different DL architectures and training parameters (Supplementary Fig. 1b,c). The best performing model was based on ResNet50, with dropout 0.2 and initial learning rate of 1e-4. Using 5-fold cross-validation, the mean k-fold achieved tile-level accuracies of >90% in training and validation, with consistent performances across folds, and no evidence of overfitting. The final model was re-trained on the entire training cohort (187 cSCC) for 40 epochs. The optimal threshold for area selection was determined based on accuracy compared to the histopathologist-annotated ROI in the training cohort. A tile prediction score of 0.65 was selected as cutoff for selection.

**Fig. 2:**
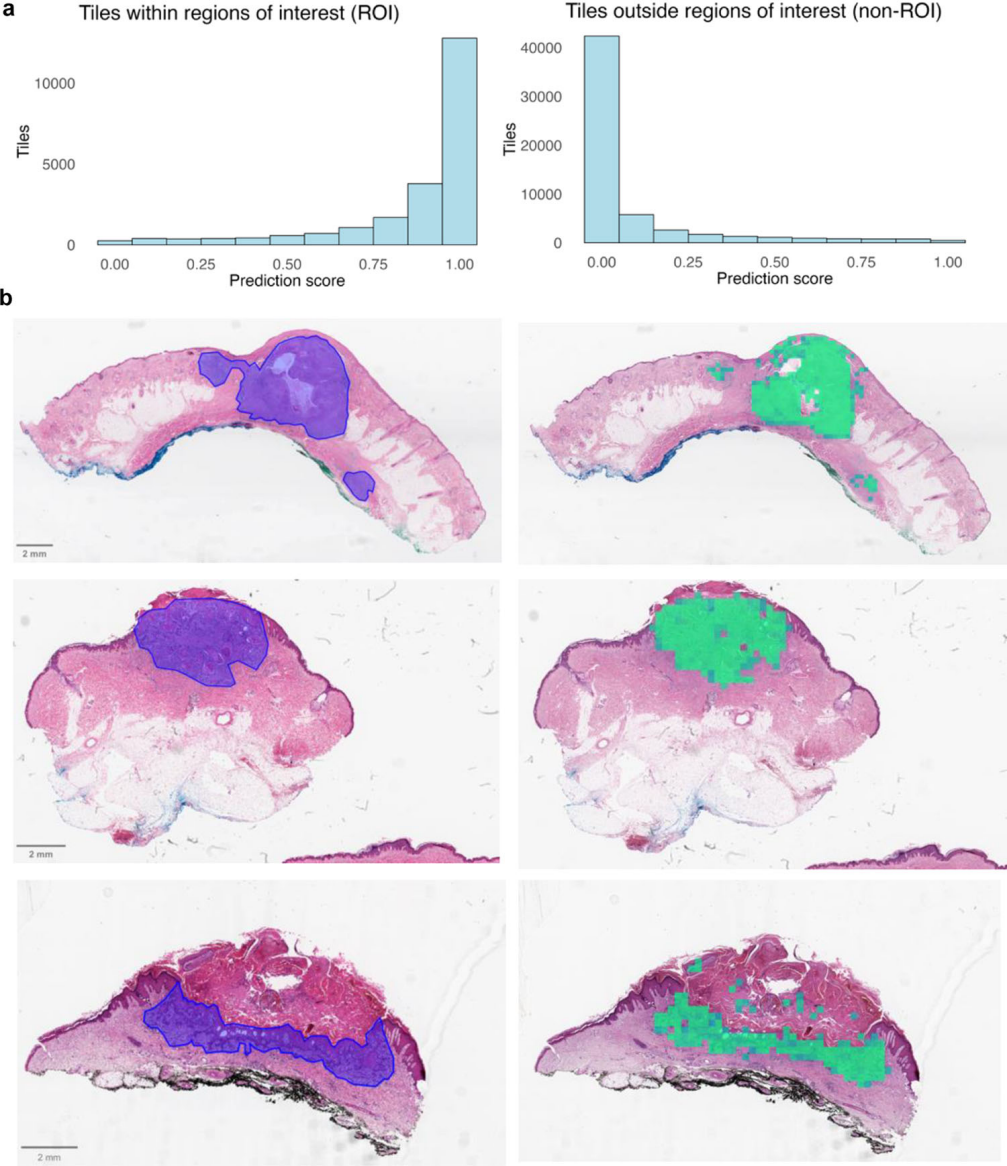
Model 1 (area selection) evaluation on the testing cohort. **a** Histograms showing tile-level predictions for tiles within the pathologist-annotated regions of interest (ROI) and for tiles outside annotations (non-ROI) in the testing cohort (*n* = 40 cSCC). A prediction score close to 1 indicates high confidence that the tile belongs inside the ROI, while a prediction score close to 0 indicates high confidence of belonging outside the ROI. **b** Whole slide images with the pathologist-annotated ROI in blue and corresponding heatmaps with Model 1-selected tiles in green.

To evaluate the performance of Model 1, predictions were generated on 80,985 tiles from the WSI in the testing cohort (*n* = 40 cSCC), which were not previously seen by the model. The 22,389 tiles within the histopathologist-annotated ROI had a median (IQR) prediction score of 0.97 (0.84–0.99), whereas the 58,596 outside the ROI tiles had a median prediction score of 0.01 (6e-4–0.07) (Fig. 2a).

As determined in the training phase, tiles with a Model 1 prediction score ≥0.65 were classified as ‘ROI’, and tiles with lower scores were classified as ‘non-ROI’. Using this predefined threshold, Model 1 achieved an AUC of 0.97 (95% CI 0.97-0.98) in identifying ROI compared to pathologist annotations. Visual inspection of heatmaps by an expert dermatopathologist (HR) confirmed that all the relevant areas were adequately included across all WSI, with negligible inclusion of non-tumour regions (Fig. 2b).

### Development of Model 2 – metastasis risk prediction

For training Model 2, a total of 129,187 ROI tiles were obtained from pathologist-annotated WSI of 172 cSCC meeting inclusion criteria: 80,380 tiles from metastasising (*n* = 64) and 48,807 tiles from non-metastasising (*n* = 108) cSCC. Tumour size varied, with a median (IQR) of 1064 (555–1634) and 317 (148–591) tiles per metastasising and non-metastasising cSCC, respectively. To avoid overfitting to larger tumours, 500 tiles were randomly selected per tumour, resulting in 27,920 and 32,711 tiles from metastasising and non-metastasising tumours, respectively. Tile labels were inherited from tumour-level labels, as either ‘Metastasising’ or ‘Non metastasising’.

Using KerasTuner, the best performing model was based on ResNet50, pretrained on Imagenet, with initial learning rate 1e-4, batch size 64, dropout 0.2, sigmoid activation function in the last dense layer, binary cross-entropy as a loss function, and ADAM algorithm for optimisation. Comparisons to additional architectures (Inception, Resnet 101, ResnetV2), learning rates, tile sizes (256 × 256 pixels), omission of colour normalisation, or no pre-training on Imagenet did not improve model performance (Supplementary Fig. 1c). Additionally, the dual model cSCCNet outperformed a single model, based on all tiles of the entire WSI (Supplementary Fig. 1d–f).

**Fig. 3:**
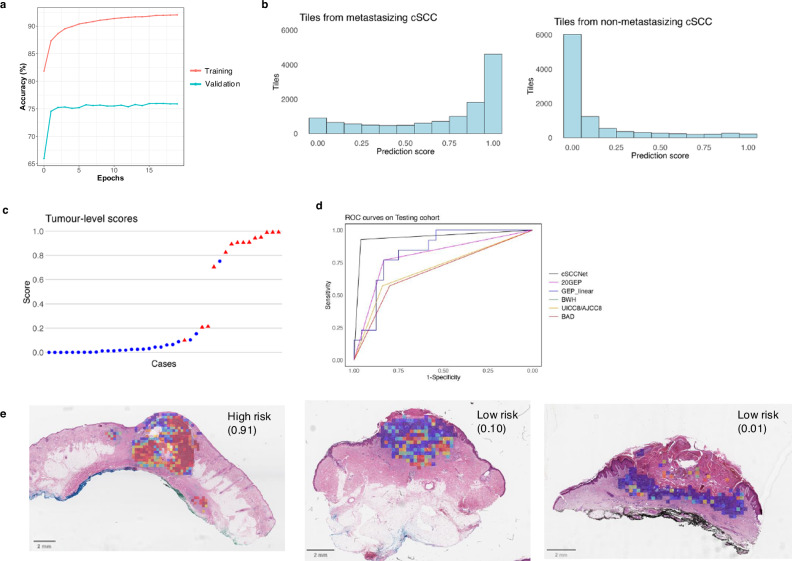
Model 2 (metastasis risk prediction) training and evaluation. **a** Mean five-fold cross validation curve for Model 2 on the training cohort (n = 172) after 20 epochs, with training accuracy in red and validation accuracy in blue. **b** Histograms showing Model 2 tile-level predictions for tiles from metastasising and non-metastasising tumours in the testing cohort (*n* = 40). A prediction score close to 1 indicates high confidence that the tile belongs to a high-risk (metastasising) tumour, while a prediction score close to 0 indicates high confidence of belonging to a low-risk (non-metastasising) tumour. **c** Tumour-level aggregate scores for metastasising (red triangles) and non-metastasising primary cSCC (blue circles) in the testing cohort. Aggregate scores >0.20 represent ‘high-risk’ tumours. **d** Receiver operating characteristic (ROC) curves for the different risk stratification tools on the testing cohort, including: cSCCNet (black), the 20-gene expression profile model derived from k-nearest neighbours analysis (20GEP, pink) and linear predictor (GEP_linear, blue), Brigham and Women’s Hospital classification (BWH, not visible due to overlap with UICC8/AJCC8), the 8^th^ edition Union for International Cancer Control/American Joint Committee on Cancer staging manual classifications (UICC8/AJCC8, orange), and the British Association of Dermatologists’ cSCC guidelines (BAD, red). **e** Representative heatmaps, with Model 2 tile scores converted to colour using a blue to red scale for scores 0-1 (low to high-risk). The tumour-level aggregate scores (of ROI tiles selected by Model 1 and after removal of tiles with borderline scores) are displayed on the top right corner of each case, with median scores >0.20 representing ‘high-risk’ tumours. Model 1 results for these cSCC were shown in Fig. 2b.

To assess generalisability, five-fold cross-validation was performed using the best performing model. The mean k-fold achieved tile-level accuracies of 0.92 for training and 0.76 for validation after 20 epochs (Fig. 3a and Supplementary Fig. 2a, b). Following five-fold cross-validation, the final model was re-trained on the entire training cohort (172 cSCC) for 20 epochs (Supplementary Fig. 2c).

Next, we used the training cohort to select a threshold for Model 2. Median (IQR) tile scores were 0.99 (0.88–1.00) and 0.01 (1e-3-0.07) for tiles from metastasising and non-metastasising cSCC, respectively. To select a tumour-level threshold, various aggregate scores were compared. Excluding tiles with borderline scores (0.3–0.7) achieved greater separation between the two groups^26^. A median tile score >0.2 achieved 99% accuracy (correct for all training cases, except one non-metastasising cSCC). Applying both models in series in the training cohort, with ROI tiles selected by Model 1 analysed by Model 2, achieved 98% tumour-level accuracy in predicting which tumours metastasised (correct for 63/64 metastasising and 106/108 non-metastasising tumours, Supplementary Fig. 3a–d).

cSCCNet performance was next evaluated on the testing cohort (*n* = 40 cSCC) using both models applied in series and the predefined thresholds (Threshold 1: scores ≥0.65; Threshold 2: median >0.2 after excluding tiles with Model 2 scores of 0.3-0.7). Model 1 selected 12,295 tiles from metastasising primaries and 9,856 tiles from non-metastasising primaries. Model 2 predictions had median (IQR) values of 0.87 (0.45-0.99) for tiles from metastasising primaries and 0.02 (1e-3-0.17) for tiles from non-metastasising primaries (Fig. 3b). cSCCNet correctly classified 38/40 cases: 13/14 metastasising cSCC were classified as high-risk and 25/26 non-metastasising cSCC were classified as low-risk by the model (Fig. 3c). Data from most cases (n = 38/40) was available for comparison with clinicopathologic classifications, including UICC8/AJCC8, BWH and British Association of Dermatologists’ cSCC guidelines (BAD), and with our published 20-GEP test^14^. cSCCNet achieved an AUC of 0.95 (95% CI 0.87-1), exceeding that of the 20-GEP test (AUC 0.80, 95% CI 0.67–0.94), although this difference was not significant. cSCCNet significantly outperformed all clinicopathologic classifications (AUC range: 0.69-0.71, DeLong test, *p* < 0.006) (Fig. 3d, Table 1). On comparison, using data from the whole cohort (172 training and 40 testing samples), cSCCNet maintained superior performance in predicting metastasising and non-metastasising cases (AUC = 0.98), followed by the 20-GEP signature (0.86), whilst the clinicopathologic classifications had inferior performances (0.74–0.78) (Supplementary Fig. 4a).

**Table 1:** Predictive performance of cSCCNet, 20-GEP test and clinicopathologic classifications on the testing cohort (n = 40)

		AUC	Accuracy	Specificity	Sensitivity	NPV	PPV	TN	TP	FN	FP	AUC (n = 35)
**cSCCNet**		0.95 (0.87–1)	95%	96%	93%	96%	93%	25	13	1	1	0.94 (0.85–1)
**20-GEP**		0.80 (0.67–0.94)	81%	83%	77%	87%	71%	20	10	3	4	0.80 (0.65–0.94)
**UICC8/AJCC8**		0.71 (0.55–0.86)	74%	84%	57%	78%	67%	21	8	6	4	0.68 (0.52–0.84)
**BWH**		0.71 (0.55–0.86)	74%	84%	57%	78%	67%	21	8	6	4	0.68 (0.52–0.84)
**BAD**	High/Very High	NA	64%	44%	100%	100%	50%	11	14	0	14	NA
	Very High only	0.69 (0.53–0.84)	72%	80%	57%	80%	62%	20	8	6	5	0.66 (0.50–0.83)

Performance in predicting risk of cSCC metastasis, based on cSCCNet prediction, the 20-GEP model outcome derived from k-nearest neighbours analysis, the 8th edition Union for International Cancer Control/AJCC staging manual (UICC8/AJCC8) stages T3 or higher, BWH stages T2b or higher, and the British Association of Dermatologists’ cSCC guidelines (BAD) based on ‘High/Very high’ risk or ‘Very High’ risk only. The GEP signature and clinicopathologic classifications were not available for all tumours; the column on the right shows AUC results for the 35 tumours with complete data. AUC: area under the receiver operating characteristic curve; FN: false negatives; FP: false positives; NPV: negative predictive value; PPV: positive predictive value; TN: true negatives; TP: true positives. The 95% confidence intervals are in brackets.

Upon investigating other benchmarking measures, cSCCNet achieved the highest accuracy (95%) and specificity (96%) in predicting which tumours metastasised in the testing cohort, outperforming the other risk stratification tools (Table 1). cSCCNet reached 93% sensitivity, superior to all other criteria except BAD ‘High/Very high’ risk category. The Pearson correlation between the 20-GEP test and cSCCNet score was 0.66 (*p* = 6e-6) for 37 cases, indicating a potential association between histopathological and molecular features (Supplementary Fig. 4b, c). On univariate analysis, features predictive of metastasis (*p* < 0.05) in the testing cohort included the cSCCNet classification, 20-GEP, UICC8/AJCC8, BWH, BAD Very High risk grade, tumour diameter, differentiation, thickness, and presence of lymphovascular invasion. Age, sex, site of primary cSCC, and presence of perineural invasion were not statistically significant in the testing cohort; however, all were significant (*p* < 0.05) when assessed in the entire cohort (*n* = 212), suggesting an impact of sample size (Supplementary Fig. 4d–i). On multivariate analysis, cSCCNet was an independent predictor of metastasis from UICC8/AJCC8 (multivariate Wald test, *p* = 002) and BWH (*p* = 6.9e-4) (Supplementary Fig. 4j,k).

### Evaluation of model training strategy using centre-split cross-validation

To evaluate whether inter-centre variability affects model performance, we trained a risk prediction model (Model 2) on cases from only three study centres, and tested this model on the fourth centre (i.e., not seen during training).

Two centre-split experiments were performed: Model BCD (trained on centres B, C, and D, and tested on centre A) and Model ABD (trained on centres A, B, and D, and tested on centre C). Results are presented in Supplementary Fig. 5. Although performance declined using the centre-split models, both models retained reasonable predictive ability when testing on entirely unseen centres, especially Model ABD, with accuracy of 73% and sensitivity of 85%, with poorer specificity of 58%. Of note, the training cohorts in the centre-split models were very unbalanced, with a lower proportion of metastasising cases likely contributing to poorer performance. These findings support our training strategy for cSCCNet, which incorporates cases from all four centres to optimise data diversity and model generalisability.

### Histopathology and immunohistochemistry (IHC) analysis to improve model explainability

Heatmaps of Model 2 outputs were interrogated for both metastasising and non-metastasising cases. Significant intratumoral heterogeneity was observed in some cSCC, with both low- and high-risk areas present within the same WSI (Fig. 3e). An expert dermatopathologist (HR) reviewed the most predictive tiles in correctly classified cases, with review of all H&E in the testing cohort and the available IHC slides. This preliminary observational analysis was not aimed to fully explain model scores, but rather to explore whether histopathological features may explain low or high scores across the cohort.

**Fig. 4:**
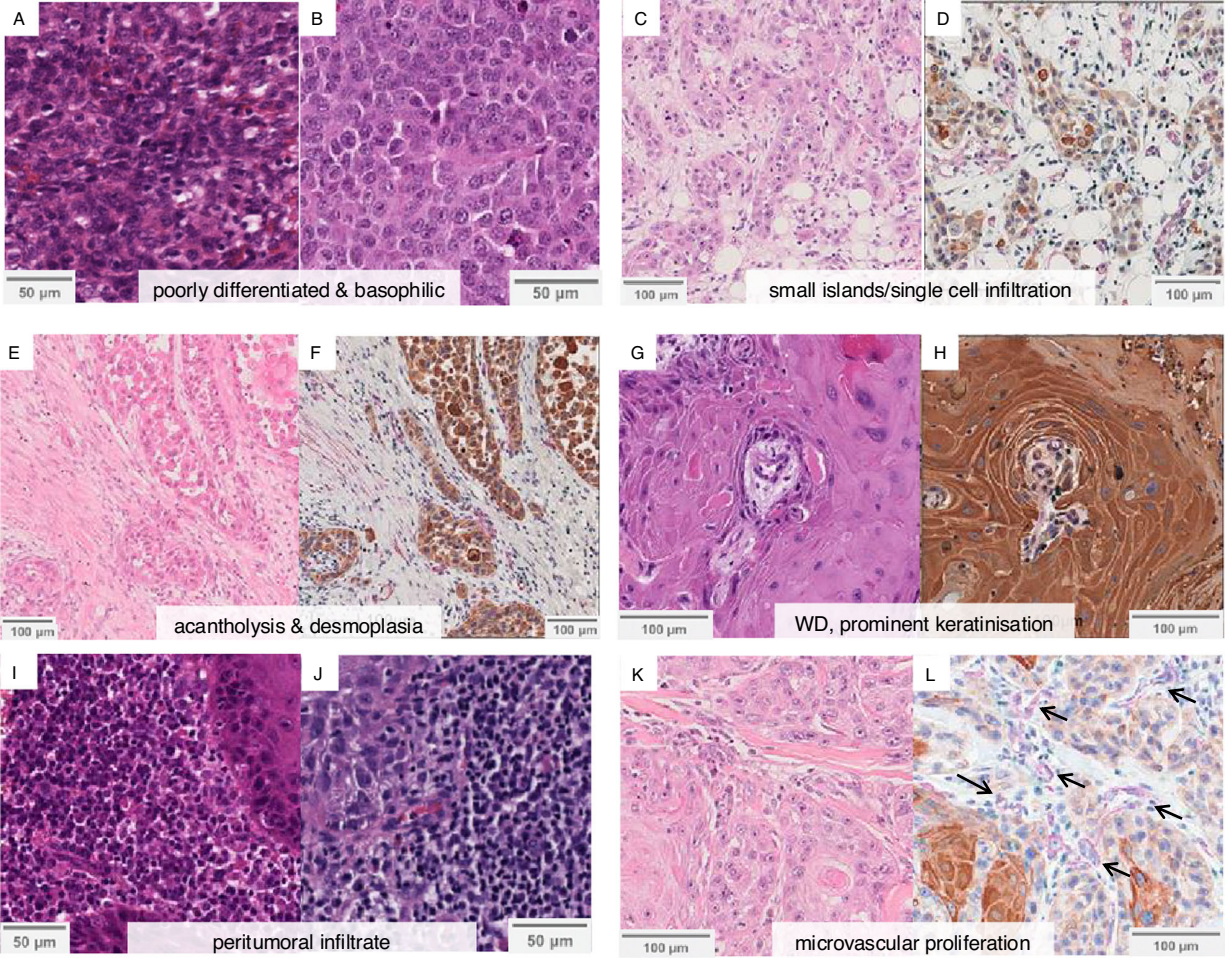
Histopathological review contributes to model explainability. Representative areas scored as ‘high-risk’ for metastasis by cSCCNet are shown, including: poorly differentiated carcinoma with deeply basophilic staining, secondary to large nuclei and scant cytoplasm (**A** and **B** haematoxylin and eosin-stained, H&E), poorly differentiated carcinoma composed of small islands and infiltrative strands (**C** H&E and **D** immunohistochemistry, IHC), and acantholytic carcinoma with discohesive invasion pattern and desmoplasia (**E** H&E and **F** IHC). Representative ‘low-risk’ areas show well differentiated cSCC and prominent keratinisation (**G** H&E and **H** IHC), and a dense peritumoral infiltrate (**I** and **J** H&E). Microvascular proliferation often obtained high scores (**K** H&E and **L** IHC, with arrows indicating blood vessels). In the IHC images, AE1/AE3-positive cells (keratinocytes) are stained brown with DAB, CD3-positive cells (T lymphocytes) are stained green, and αSMA-positive cells (cancer-associated fibroblasts, tumour stroma and cells surrounding blood vessels) are stained red. αSMA: alpha-smooth muscle actin; cSCC: cutaneous squamous cell carcinoma; WD: well differentiated.

The model consistently assigned high scores (indicating higher risk) to areas of poorly differentiated carcinoma, which were often characterised by deeply basophilic staining secondary to large nuclei and scant cytoplasm (Fig. 4A, B). Additionally, areas with necrosis, single cell infiltration (Fig. 4C, D), acantholysis, or prominent desmoplasia surrounding carcinoma (Fig. 4E, F) often received borderline or high scores. Conversely, low scores (indicating lower metastatic risk) were assigned to regions containing predominantly near-normal epidermis, well-differentiated carcinoma (Fig. 4G, H), lymphocyte aggregation at the tumour edge (peritumoral infiltrate) (Fig. 4I, J), or cystic regions. Regions with dense, deeply eosinophilic stroma and keratin (Fig. 4G, H) were also consistently assigned low scores. Of note, tumour areas containing abundant blood vessels (Fig. 4K, L) often received high scores; however, it was unclear whether vascularisation itself was being recognised as a poor prognostic feature or whether the vessels were mimicking poorly differentiated carcinoma. Certain model predictions could not be fully explained, suggesting that cSCCNet may be detecting features beyond known histopathological risk factors.

**Fig. 5:**
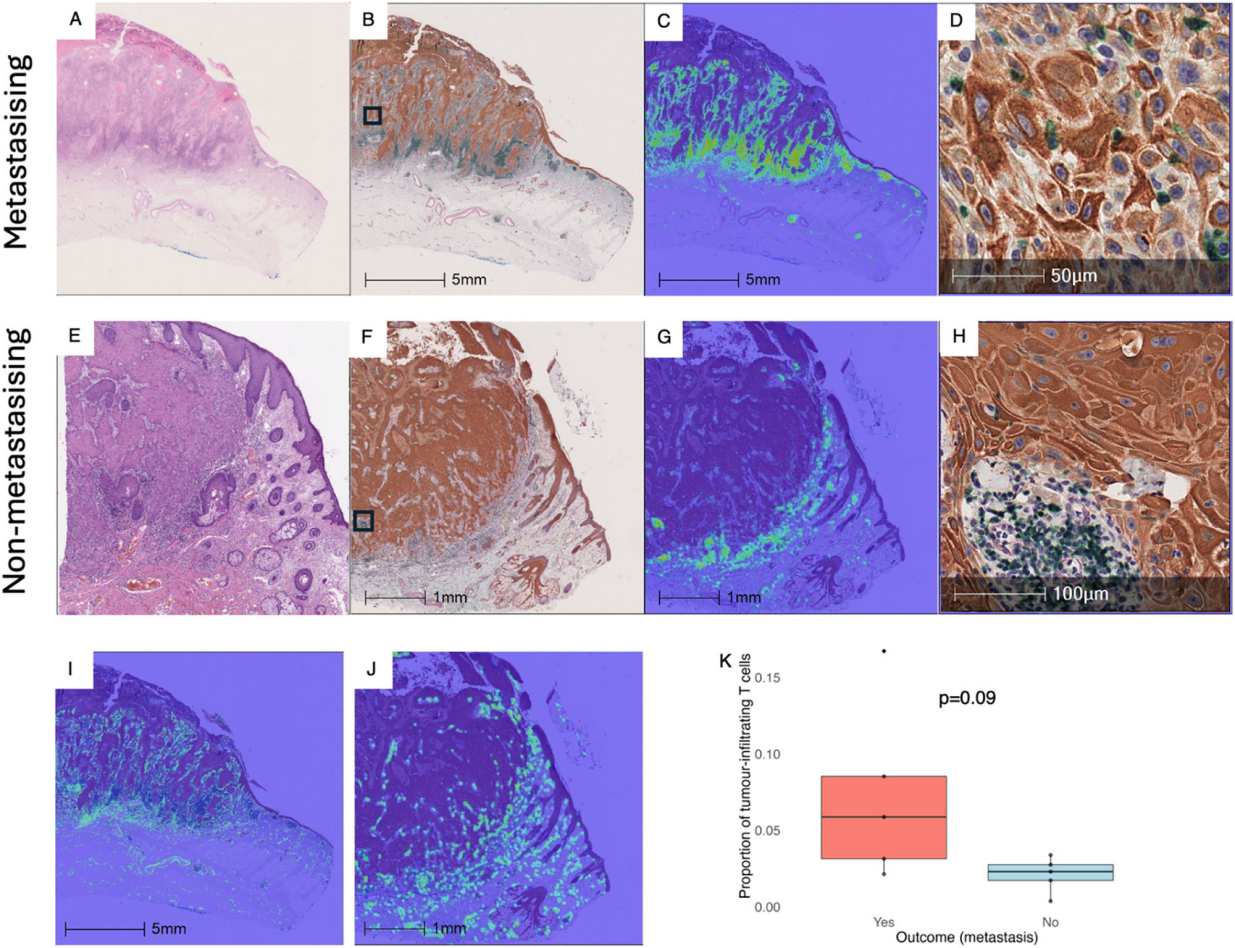
Multiplex immunohistochemistry (IHC) of additional five metastasising vs five non-metastasising cSCC. Multiplex IHC was performed on 10 cSCC, with two representative cases shown: a metastasising cSCC (**A–D** and **I**) and a non-metastasising cSCC (**E–H** and **J)**. The H&E-stained slides are shown in **A** and **E**. Multiplex IHC was performed on adjacent sections (**B** and **F**; the black squares correspond to the high power images in **D** and **H**) with AE1/AE3-positive cells (keratinocytes) stained brown with DAB, CD3-positive cells (T lymphocytes) with green, and αSMA-positive cells (cancer-associated fibroblasts, tumour stroma and cells surrounding blood vessels) with red. T-cell density heatmaps are shown, with CD3-positive cells highlighted green (**C** and **G**). High-power IHC images show representative areas of T-cell infiltration within a metastasising cSCC (intratumoral infiltration, **D**) and peritumoral infiltration at the deep tumour edge of a non-metastasising cSCC (**H**). αSMA density heatmaps are shown with αSMA positive cells highlighted green (**I** and **J**). **K** Analysis of multiplex IHC showed greater proportions of tumour-infiltrating T cells within tumour regions (intratumoral infiltration) in metastasising cSCC compared to non-metastasising cSCC. Box-plot elements: centre line, median; box limits, upper and lower quartiles; whiskers, 1.5x interquartile range; points, individual data points; x-axis: metastasising (Yes) vs non-metastasising (No) cSCC; y-axis: proportion of tumour-infiltrating T cells, defined as the number of CD3-positive cells divided by the total number of cells (of any type) within tumour regions, as calculated using the HALO-AI classifiers. The Mann-Whitney U test was used to compare the two groups (p = 0.09). αSMA: alpha-smooth muscle actin; cSCC: cutaneous squamous cell carcinoma; H&E: haematoxylin and eosin; IHC: immunohistochemistry.

Multiplex IHC was performed on further 5 metastasising and 5 non-metastasising cases, allowing improved separation of different cell types and more detailed assessment of cell type composition in individual tiles (Fig. 5). Keratinocytes were identified by anti-AE1/AE3 (stained with DAB). T lymphocytes were highlighted with anti-CD3 (stained in green). The third cell marker, αSMA (alpha-smooth muscle actin, in red), is expressed by several cell types, including cancer-associated fibroblasts, tumour stroma, and by cells surrounding blood vessels, including capillaries. Qualitative analysis revealed greater T cell infiltration within metastasising tumours (i.e., intratumoral infiltrate) (Fig. 5C, D) compared to non-metastasising tumours (Fig. 5G, H). Quantitative analysis using HALO-AI estimated the median (IQR) proportion of CD3-positive cells within tumour regions (tumour-infiltrating T cells) as 6% (3–9%) within metastasising cSCC and 2% (2–3%) in non-metastasising cSCC (Fig. 5K) although this difference did not reach statistical significance (Mann-Whitney U test, *p* = 0.09), likely due to the small sample size.

### Review of incorrect cases

Two cases in the testing cohort were misclassified by the model. One non-metastasising scalp cSCC received a high model score (0.75, Supplementary Fig. 6a). On histopathological review, it was poorly differentiated, invaded beyond the subcutis, and was classified as high-grade by UICC8/AJCC8 (T3) and BWH (T2b). Examination of cSCCNet heatmaps revealed that Model 1 had failed to select >60% of the ROI, and that the small number of tiles passed to Model 2 were deeply basophilic. In this case, we attributed the misclassification to sampling bias and difficulty of the case. One metastasising pinna cSCC with incomplete excision margins received a low model score (0.10, Supplementary Fig. 6b). The majority of the tumour was moderately-differentiated with good keratinisation; however, there was extension beyond cartilage. A small area of poorly differentiated carcinoma was present and was correctly classified as ‘high-risk’ by the model. It was staged UICC8/AJCC8 T3 and BWH T2b. Of note, this tumour had initial incomplete margins and underwent re-excision.

## Discussion

cSCCNet is an automated DL tool, able to predict metastatic risk from digitised H&E slides of primary cSCC. In this study, cSCCNet outperformed commonly used clinicopathologic classifications, achieving the highest AUC and accuracy for predicting cSCC metastasis. As expected, the dual-model cSCCNet, which uses automatically-selected ROI, outperformed models based on the entire WSI by excluding ‘noisy’ data prior to risk prediction.

To avoid introducing bias and aware of the limitations of currently-used histopathological features, we deliberately did not predefine which morphological features the model should focus on when predicting metastatic risk. Instead, the model was presented with all tumour tiles (ROI) and allowed to independently learn and extrapolate the morphological patterns most predictive of metastasis. Review of model output, aided by multiplex IHC, showed that the degree of tumour differentiation, acantholysis, desmoplasia, and the spatial localisation of lymphocytes relative to tumour regions were important prognostic factors, aligning with existing literature on cSCC progression^27^. Well-differentiated cSCC and prominent keratinisation are associated with low metastatic potential, whilst poor differentiation favours tumour progression and metastasis^28^. Desmoplasia is also widely recognised as a high-risk feature in cSCC, although its identification and scoring can be challenging^29^. The value of acantholysis in predicting biological outcomes is still equivocal, and the significance is yet to be firmly established^30^.

Peritumoral infiltration was consistently assigned low-risk scores by the model, consistent with previous studies indicating that high peritumoral inflammation is a good prognostic factor^31,32^. We also observed a trend of greater intratumoral infiltration in metastasising cases compared to non-metastasising cases on IHC. This requires validation in larger cohorts, and further investigation into which T cell subtypes play a key role, but may be due to the presence of regulatory T cells (Tregs), including OX40 + T regs, in the infiltrate, which has been reported previously by our group to suppress tumoral effector T cells and associate with cSCC metastases^33^. Furthermore, tumour-infiltrating exhausted CD8 + T cells have also been shown to associate with adverse outcomes in many cancers^34,35^. Tumour vascularisation, which has not been extensively studied in cSCC, may also be a feature recognised by cSCCNet and warrants further investigation.

A robust and generalisable prognostic model relies on having a diverse training cohort^36^. This is relevant in cSCC, where there is great diversity in tumour and patient factors^28^. Inter-centre differences in slide processing and image acquisition result in additional data variability^37^. Evaluating our model training strategy using centre-split cross-validation supported our model training strategy for cSCCNet. This study utilises a large primary cSCC WSI dataset, comprising 172 cases from four different institutions for model training; one of the largest reported to date for this task. In comparison, Knuutila et al.’s final model used a single-centre cohort of 81 cSCC, split into training and testing via 4-fold cross-validation^21^. Coudray et al. trained their model on 163 patients from three institutions^22^. Pisula et al^23^. report a dataset comprising 243 patients from three centres, and the 65% training split suggests approximately 158 patients were used for model training. An additional methodological strength of our current study lies in efforts to ensure the accuracy of outcome labels, by ensuring adequate follow-up duration for non-metastasising cSCC.

Additional steps taken to increase generalisability included training on cSCCs from four centres, using transfer learning and data augmentation techniques, and evaluation of model performance on cases it had not seen before. Whilst class balance in this study does not reflect the real-world proportion of metastatic cSCC, enrichment with primaries that metastasised was deliberate, to help the model better learn the patterns associated with high-risk cSCC.

An advantage of cSCCNet over standard clinicopathologic classifications is the elimination of inter-rater variability, which can occur due to differences in reporting of differentiation, depth or perineural invasion^38^. A further strength is that, as it is based on standard histopathology slides, this analytical tool could be incorporated into existing histopathology workflows. Unlike many models, cSCCNet does not require time-intensive manual annotation by histopathologists, as it automatically selects the area for analysis, reducing noise by excluding non-tumour regions. Heatmap visualisations contribute to quality control, ensuring that all relevant tissue is being selected, as well as to model interpretability and may provide insights into potential drivers for metastasis.

Whilst this study focused on UV-related cSCCs, which are the most common aetiology, we aim to evaluate this model in cSCCs with distinct aetiologies, such as arsenic- or HPV-induced cSCCs, or in tumours arising within chronic wounds or areas of inflammation. A further limitation for generalisability is that we have not trained the model on tumours that have recurred locally without evidence of regional or distant metastases, and future iterations of the model will aim to include these.

Review of incorrectly classified cases revealed that cSCCNet requires full excision specimens or there is risk of misclassification. Although cSCCNet does not measure the depth of invasion, we observed that the deep tumour margins often contain important prognostic information, and this highlights the need to include complete tumours for more accurate stratification. ROI annotations for model training were performed by a single expert dermatopathologist, and we acknowledge that inter-observer variation in tile selection is possible; however, minor variations in ROI selection are unlikely to affect final cSCCNet predictions, which are based on tumour-level aggregate scores. In addition, heatmaps provide a transparent and interpretable way to verify automated area selection.

cSCCNet is a result of an ongoing multidisciplinary collaboration. Further fine-tuning and validation in external cohorts and important subgroups are planned, such as in patients with cSCC of non-UV aetiologies, immunosuppressed individuals, international populations, and centres using diverse staining protocols and scanners. This will also make the model more generalisable and robust to inter-patient and inter-centre differences. Evaluation in a large, prospective multi-centre test cohort using federated learning to facilitate multi-centre collaboration is now planned^23,37^.

Future work is focused on developing improved explainability methods^39^, comparing to foundation models and newer approaches, incorporating measures of prediction certainty and robustness, and integrating the model into existing histopathology workflows. There is also potential for combining digital pathology analysis with clinical and genetic data into a multimodal DL tool^40^. Future studies would be needed to confirm that cSCCNet is of benefit in informing use of interventions aimed at reducing risk of metastasis, such as a randomised controlled trial evaluating cSCCNet in risk stratification for adjuvant cSCC treatment.

Use of digital pathology is becoming widespread, making AI-based technologies more accessible^41^. Multidisciplinary collaborations between clinicians, AI experts and patients will be critical in developing these models into useful and effective clinical tools. We envision using cSCCNet to support skin cancer teams in clinical decision making, as combining digital pathology analysis with known risk factors may improve patient stratification. In addition to improving outcomes for cSCC patients, this could lead to more efficient use of healthcare resources for this very common cancer.

## Methods

### Patient selection and data collection

This study was conducted in accordance with the Declaration of Helsinki and was approved by the NHS Human Research Authority (IRAS 266559, ‘Diagnostic marker panel development for progression in skin cancer’; ethics reference^20^:/WM/0018; West Midlands, Solihull Research Ethics Committee). Participant consent was not required as the study used routinely collected information only. The CLAIM and TripodAI checklists were followed^42,43^. The patient selection criteria have been published and were aimed at acquiring a diverse and representative sample for model development^14^. Briefly, four UK pathology centres (Glasgow’s Queen Elizabeth Hospital, Cheltenham General Hospital, Southampton General Hospital and Barts Health NHS Trust) identified patients with primary cSCC with pathological evidence of metastasis or with primary cSCC that had not metastasised within three years of excision. The minimum of three years of follow-up ensures that over 90% of metastases are captured^4^.

Immunosuppressed patients were excluded. Hospital electronic patient records for all patients were reviewed, and demographic and outcome data were recorded, including sex, age at cSCC diagnosis, ethnicity, whether the patient had cSCC locoregional or distant metastasis, and time to last follow-up. H&E sections were digitally scanned by a Leica scanner and Aperio software to obtain WSI at 20x magnification and saved in SVS format. Images were reviewed centrally by expert dermatopathologists (PC, WR) and primary tumours histologically staged using UICC8/AJCC8, BWH and BAD classifications; these are referred to as ‘clinicopathologic classifications’ in this paper^8,44^. We used the UICC8 criteria as this is used routinely in the UK, but we modified it to use the AJCC8 definition of perineural invasion (nerve ≥0.1 mm diameter or deeper than the dermis), as recommended by the Royal College of Pathologists^45^; this modification effectively makes the two criteria equivalent. We excluded cases if the WSI did not contain invasive cSCC (i.e., cSCC-in situ with no evidence of invasion), >50% of the tumour region had artefact/blurring, or if an H&E-stained section was not available. The final cohort is therefore slightly different from our published study^14^, which relied on RNA quality rather than WSI availability. All cSCC were treated with standard excision rather than Mohs surgery, allowing for more complete tumour visualisation in WSI.

A total of 227 primary cSCC were included. All patients had one cSCC, except one patient who had two primary cSCC. Four tumours had two WSI per tumour and all other tumours had one WSI per tumour. Fifteen primary cSCC did not meet the full inclusion criteria (nine were immunosuppressed, four had incomplete follow-up data, and two had local recurrence only); these were used in training Model 1 (area selection) only. The remaining 212 tumours were randomly split using an 80:20 ratio, with 172 cSCC (64 metastasising and 108 non-metastasising) for training and 40 cSCC (14 metastasising and 26 non-metastasising) for testing. The training:testing split was random and stratified by tumour outcome and contributing centre (Supplementary Fig. 1a). Baseline demographic and histopathological characteristics are summarised in Supplementary Table 1. Median age was 80 years, and 68% were male. Ethnicity was available from only one centre, where 80/84 (95%) of patients were white. There were no statistically significant differences in baseline characteristics between the two groups.

### Image tile generation and pre-processing

Our image pre-processing pipeline was developed based on the Aachen protocol^46^. All WSI were manually annotated to select the ROI, and reviewed by an expert dermatopathologist (HR) blinded to metastatic outcome. Regions with significant artefacts such as air bubbles were excluded. The WSI were tessellated into non-overlapping 512×512 pixel tiles, and all tiles from one tumour were assigned into the same (training or testing) group. Tissue annotation was implemented using QuPath version 0.5.0^47^.

Tile filtration was performed to exclude tiles with >20% empty space (defined as pixel values > 240) and blurred tiles (variation of the Laplacian <70). As slides were obtained from different laboratories, colour normalisation using the Macenko method was performed on all tiles to reduce variability from slide preparation techniques^48^. A sample of included, excluded and colour normalised tiles was reviewed for quality control (EP, BF).

Data augmentation techniques were implemented using Keras ImageDataGenerator, with each training epoch using different versions of the input images. Image transformations included rotations (up to ±360 degrees), vertical and horizontal flips, shifts in width and height (up to ±20%), zoom adjustments (up to ±30%), and alterations in brightness (0.2 to 1.5 times the original). A random sample of augmented tiles were reviewed by author EP. Augmentation was used solely for training and was not used during testing.

### Model training

The training cohort consisted in 172 primary cSCC as previously described, with an additional 15 cSCC used for training Model 1 only. The same model training pipeline was followed for Models 1 and 2 (Supplementary Fig. 1b). We first used the KerasTuner for hypertuning to select the ideal model architecture and combination of hyperparameters. We performed a systematic comparison of widely-used convolutional neural network backbones (ResNet50, MobileNetV2, VGG16, InceptionV3) and parameters, including dropout (0.1, 0.2, 0.3) and initial learning rates (1e-4,1e-5,1e-6), to select the optimal settings for the models^49^. L2 weight decay was used as an additional regularisation technique, set at 0.01 throughout training. The optimisation process using KerasTuner was facilitated by a random split of the training dataset (*n* = 187 for Model 1 and *n* = 172 for Model 2), with 80% of the cases allocated for training and 20% for validation. The training: validation split was random and stratified by tumour outcome and contributing centre. Additional important parameters were tested individually (Supplementary Fig. 1c).

After the top-performing model was selected, it was evaluated using 5-fold cross-validation to assess model robustness when presented with different datasets. The StratifiedGroupKFold function from sklearn was used to divide the training cohort into five non-overlapping groups of similar sizes (cases were randomly distributed and stratified by tumour outcome and centre). Each fold used a different group as validation. A final model was then trained on the entire training cohort (*n* = 187 for Model 1 and *n* = 172 for Model 2), optimising the use of all available training data.

To select a threshold for binary classification, the final model was used to generate predictions on the training cohort. The model assigned a score from 0 to 1 for each individual tile, corresponding to the confidence score of the tile belonging to the ROI (Model 1) or to a high-risk tumour (Model 2). The ground truth labels were the pathologist-annotated ROI (for Model 1) or metastatic outcome (for Model 2). Based on the distributions of scores between groups defined by ground truth labels, the optimal threshold was selected.

### Model evaluation

The final models and pre-selected thresholds were evaluated on the testing cohort (*n* = 40), which was not previously seen by the models. Model performance was evaluated both individually and in series. Tile-level and tumour-level performance was evaluated using predefined analyses and compared to clinicopathologic classifications, as described in Statistical analysis.

A centre-split approach was also employed to evaluate our cSCCNet training strategy, with one centre left out as an unseen test cohort. For cSCCNet, maximising the size and diversity of the training cohort was an important objective. Given that only centres A and C contributed both non-metastasising and metastasising cases, two centre-split models were trained and evaluated: Model BCD (trained on centres B, C and D, and tested on centre A) and Model ABD (trained on centres A, B and D, and tested on centre C). The models were then benchmarked against cSCCNet for predicting metastasis in unseen cases.

Heatmaps were generated based on model predictions: the tile scores assigned by the model, ranging from 0-1, were converted to colours using a matplotlib colour scale and overlain onto the original WSI. Tiles with the highest prediction scores in correct cases were retrieved, and histological features were described^50^. Heatmaps were also interrogated in incorrect cases to gain insight into model limitations.

Python code was employed for tile filtration, model development and evaluation. Analyses were performed using Queen Mary University of London’s (QMUL) High-Performance Computing cluster.

### Immunohistochemistry (IHC)

To gain further insight into cSCCNet predictions, brightfield multiplex IHC was performed on a further five metastasising and five non-metastasising cSCC using the using the Ventana Discovery ULTRA platform (Pathology Department, Barts Cancer Institute, London UK). Tissue sections adjacent to the WSI were incubated with the following antibodies: anti-cytokeratin AE1/AE3 for keratinocytes (M3515, Dako), anti-CD3 for T lymphocytes (ab11089, Abcam), and anti-αSMA (A2547, Sigma). αSMA is expressed by several cell types, including cancer-associated fibroblasts, tumour stroma, and by cells surrounding blood vessels including capillaries. Anti-CD31 staining for endothelial cells was evaluated, confirming that anti-αSMA was accurately identifying all blood vessels; anti-CD31 was excluded from the final IHC protocol. The chromogens used included: DAB for AE1/AE3, green HRP for CD3, and red HRP for αSMA. Sections were finally counter stained with haematoxylin.

IHC slides were scanned using a Nanozoomer S210 scanner at 40x magnification, and images were analysed using the HALO-AI image analysis software platform (Indica Labs Inc, London UK). A HALO-AI MiniNet convolutional neural network was trained on annotated sections of all WSI, to classify tissue into ‘tumour’, ‘epidermis’, ‘dermis’ and ‘background’. The HALO-AI object phenotyper, with embedded pre-trained nuclear segmentation, was trained to classify cells into four phenotypes: keratinocytes (DAB), T lymphocytes (green stain), blood vessels and tumour stroma (red stain), and other. All classifications were reviewed by a histopathologist (HR) to confirm their accuracy.

### Statistical analysis

Descriptive statistics were used to summarise clinical and histopathological characteristics, including median and interquartile range. Differences between the groups were assessed using the nonparametric Mann-Whitney U test for continuous variables and Fisher’s exact test for categorical variables. The primary outcome was prediction accuracy for metastasis, evaluated by the AUC and its 95% confidence interval using the DeLong method^50^. Performance was also evaluated by calculating accuracy, sensitivity, specificity, PPV and NPV^51^. For comparison, these metrics were also calculated for the 20-GEP test and for clinicopathologic classifications (UICC8/AJCC8, BWH and BAD).

The association between cSCCNet model output and 20-GEP signature was further evaluated with scatterplots and Pearson correlation. Univariate cox regression analysis and multivariate cox proportional hazards models identified factors predictive of cSCC metastasis. Statistical significance was set at 0.05 (two-sided) and statistical analysis was performed using R studio, Version 2024^52^.

## Supplementary information

Supplementary materials

## Data Availability

The datasets generated and/or analysed during the current study are partially available as cohort summaries in this published article. Digital pathology images are available from the corresponding authors on reasonable request and with permission of the study sponsor, the four participating centres, Queen Mary University of London and Cancer Research UK Scotland Institute, Glasgow.

## Code availability

The code for this study and the trained models are publicly available on Github (https://github.com/BioInforCore-BCI/SCCNet/).
