# Supplementary material for: Enhanced metastasis risk prediction in cutaneous squamous cell carcinoma using deep learning and computational histopathology
Source: NPJ Precis Oncol. 2025 Sep 2;9:308. doi: 10.1038/s41698-025-01065-7 (PMC12405460; doi:10.1038/s41698-025-01065-7)
Supplement: Supplementary file 1 — Supplementary materials [file 41698_2025_1065_MOESM1_ESM.docx]

**Supplementary Material**

**Supplementary Figure 1. Model development**


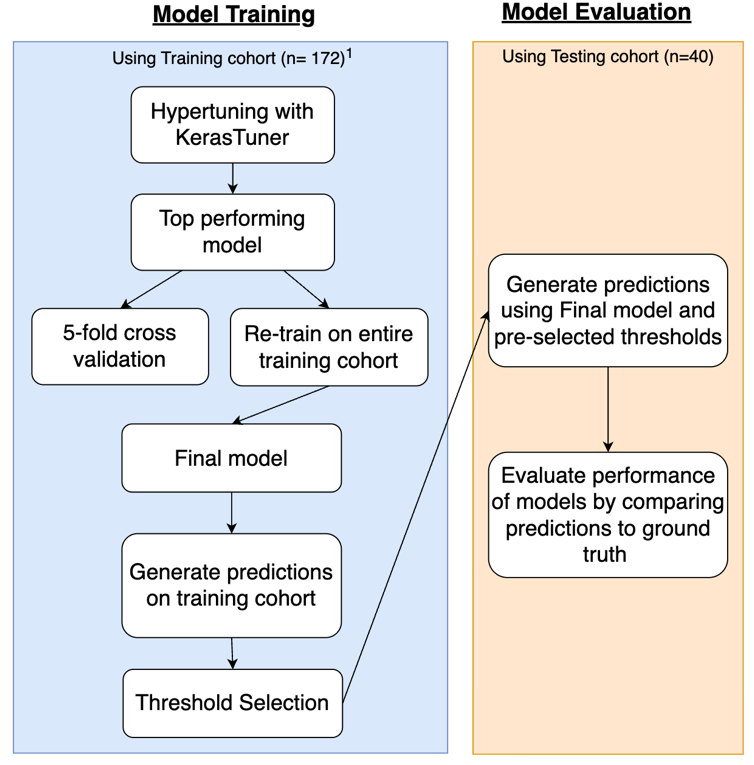


b


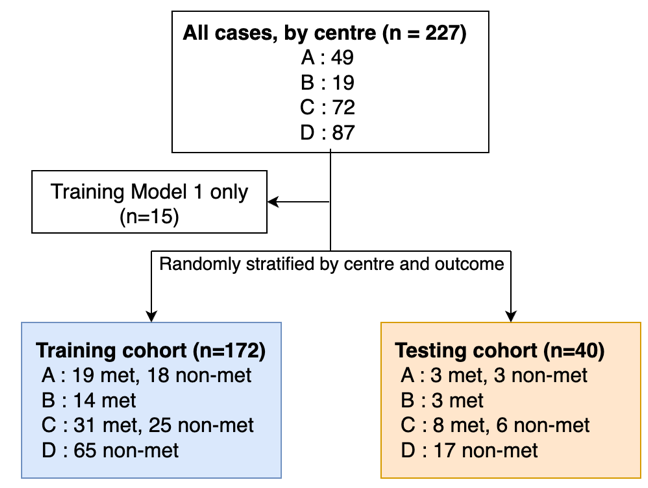


a

c

| **Parameters compared using KerasTuner:**   - Network: ResNet50, MobileNetV2, VGG16, InceptionV3 - Dropout: 0.1, 0.2, 0.3 - Initial learning rate: 1e-4, 1e-5, 1e-6 - Fixed parameters: batch size 48, epochs 20, sample cap 500 tiles/tumour, pretrained on Imagenet, tile size 512x512 pixels, decay=0.01 |
| --- |
| **Additional parameters evaluated for Model 2:**   - Networks: ResNetV2, Resnet101, InceptionResNetV2 - Batch size: 16, 24 - Initial learning rate: 1e-2, 1e-3 - Dropout: 0.5 - Not pretrained on ImageNet - Trained on entire WSI (vs limiting to Region of interest) - Tile size: 256x256 pixels - Without colour normalisation - Sample cap: 1000 tiles/tumour |


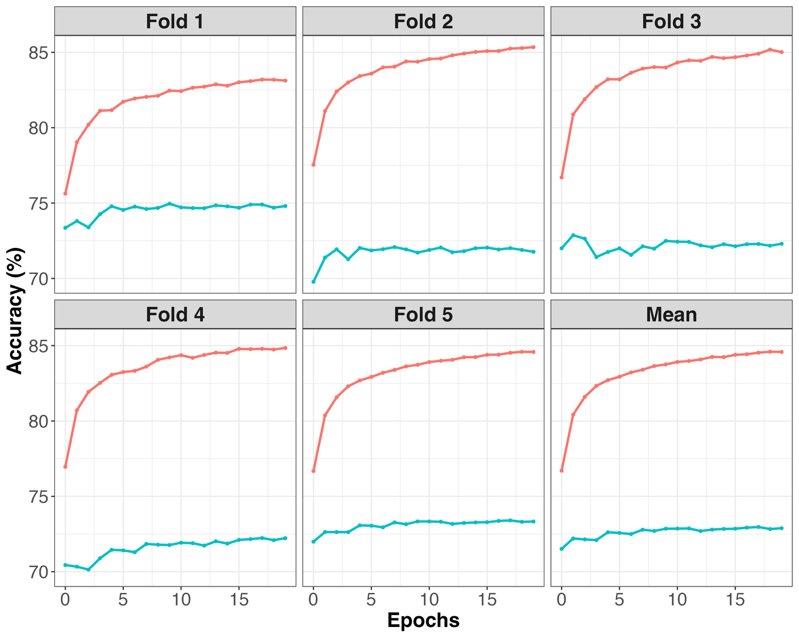


d

e

| Epochs | Training accuracy | Training loss | Validation accuracy | Validation loss |
| --- | --- | --- | --- | --- |
| 0 | 0.767 | 0.480 | 0.715 | 0.518 |
| 1 | 0.804 | 0.423 | 0.722 | 0.511 |
| 2 | 0.816 | 0.403 | 0.721 | 0.524 |
| 3 | 0.823 | 0.392 | 0.721 | 0.516 |
| 4 | 0.827 | 0.387 | 0.726 | 0.521 |
| 5 | 0.829 | 0.381 | 0.726 | 0.526 |
| 6 | 0.832 | 0.376 | 0.725 | 0.524 |
| 7 | 0.834 | 0.376 | 0.728 | 0.522 |
| 8 | 0.836 | 0.372 | 0.727 | 0.524 |
| 9 | 0.838 | 0.368 | 0.729 | 0.526 |
| 10 | 0.839 | 0.367 | 0.729 | 0.529 |
| 11 | 0.840 | 0.364 | 0.729 | 0.525 |
| 12 | 0.841 | 0.363 | 0.727 | 0.532 |
| 13 | 0.843 | 0.362 | 0.728 | 0.532 |
| 14 | 0.842 | 0.362 | 0.728 | 0.529 |
| 15 | 0.844 | 0.358 | 0.729 | 0.534 |
| 16 | 0.844 | 0.356 | 0.729 | 0.530 |
| 17 | 0.845 | 0.356 | 0.730 | 0.534 |
| 18 | 0.846 | 0.355 | 0.728 | 0.535 |
| 19 | 0.846 | 0.355 | 0.729 | 0.536 |

f

|  | **AUC**  **(95% CI)** | **Accuracy** | **Specificity** | **Sensitivity** | **NPV** | **PPV** | **TN** | **TP** | **FN** | **FP** |
| --- | --- | --- | --- | --- | --- | --- | --- | --- | --- | --- |
| **WSI model** | 0.88  (0.80-0.97) | 85% | 77% | 100% | 100% | 70% | 20 | 6 | 0 | 14 |
| **cSCCNet** | 0.95  (0.87-1) | 95% | 96% | 93% | 96% | 93% | 25 | 13 | 1 | 1 |

**a** Diagram summarising included cases: a total of 227 primary cSCC from 4 study centres were initially selected. Fifteen primary cSCC did not meet the full inclusion criteria; these were used in training Model 1 (for cSCC area selection) only. The remaining 212 tumours were randomly split using an 80:20 ratio, with 172 cSCC for model training (Training cohort) and 40 cSCC for model evaluation (Testing cohort). This training:testing split was stratified by study centre and outcome. **b** Diagram summarising the model training and evaluation pipeline followed for both models. Firstly, hypertuning using the KerasTuner was used for selection of the ideal model architecture and hyperparameters, through systematic comparison of widely-used convolutional neural network backbones and parameters. After the top performing model was selected, model robustness was evaluated using 5-fold cross-validation, and it was re-trained on the entire training cohort to obtain a final model. The final model was used to generate predictions on the training cohort to select a threshold for binary classification. Model evaluation was performed by generating predictions on the (hold-out) testing cohort, and comparing model predictions to ground truth. The ground truth for Model 1 was pathologist-selected regions of interest and the ground truth for Model 2 was metastatic outcome. **c** List of parameters compared using the KerasTuner and additional important parameters evaluated individually. **d** Five-fold cross validation curves for metastasis prediction, using model based on entire WSI (WSI model), with training accuracy in red and validation accuracy in blue. The mean k-fold (bottom right curve) achieved tile-level accuracies of 0.85 for training and 0.73 for validation after 20 epochs. **e** Mean k-fold results for metastasis prediction using the WSI model (bottom right curve in Supp Fig 1d) for 20 epochs. **f** Performance of the WSI model and the final cSCCNet model on the Testing cohort (n=40). ^1^An additional 15 primary cSCC were used during all model training steps for Model 1 (area selection). AUC: area under the receiver operating characteristic curve; cSCC: cutaneous squamous cell carcinoma; FN: false negatives; FP: false positives; met: metastasizing primary cSCC; non-met: non-metastasizing primary cSCC; NPV: negative predictive value; PPV: positive predictive value; TN: true negatives; TP: true positives; WSI: whole slide image. The 95% confidence intervals are in brackets.

**Supplementary Figure 2. Model 2 training**

a


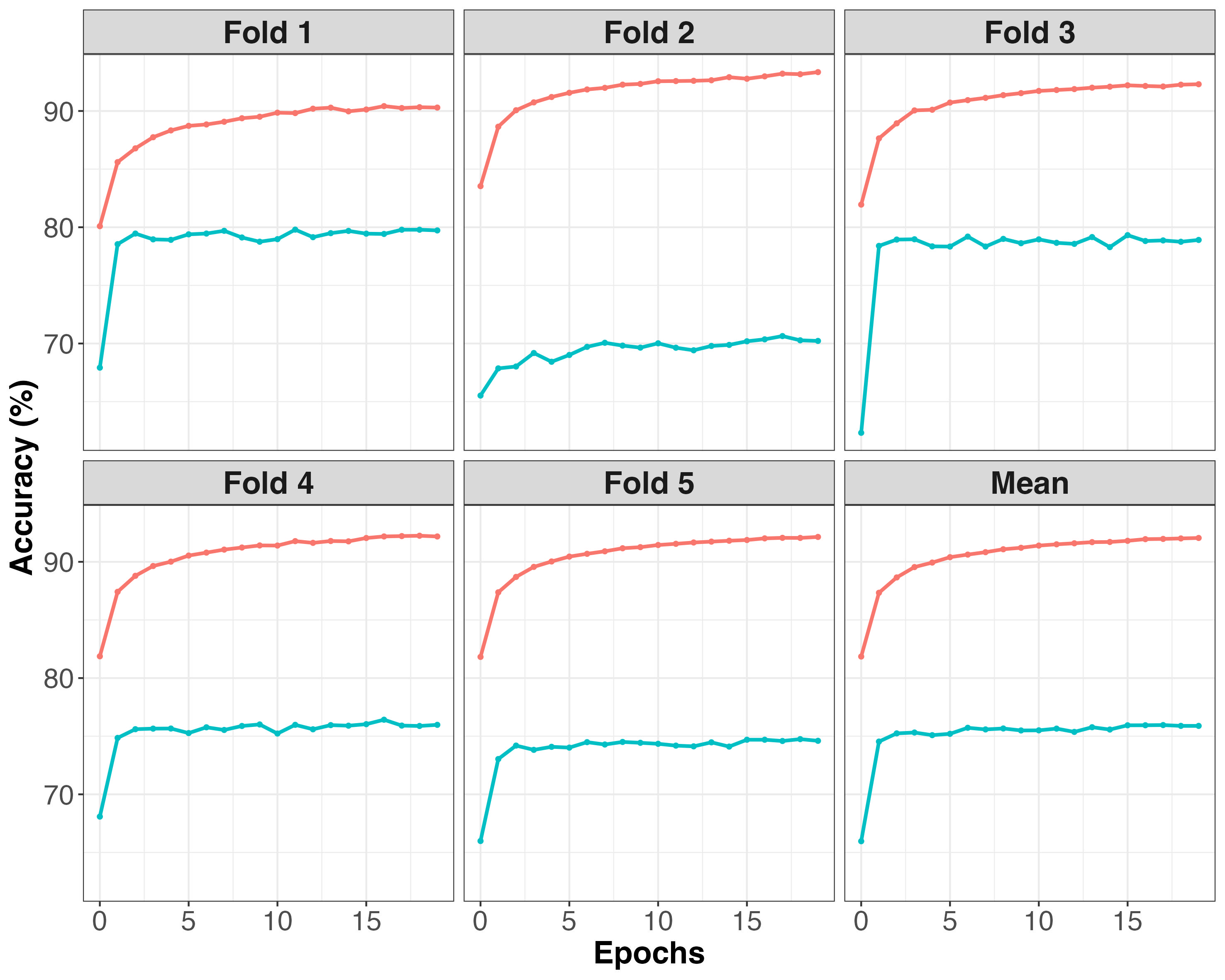


b

| Epochs | Training accuracy | Training loss | Validation accuracy | Validation loss |
| --- | --- | --- | --- | --- |
| 0 | 0.819 | 0.418 | 0.660 | 0.732 |
| 1 | 0.873 | 0.323 | 0.745 | 0.598 |
| 2 | 0.887 | 0.299 | 0.752 | 0.583 |
| 3 | 0.895 | 0.281 | 0.753 | 0.618 |
| 4 | 0.899 | 0.271 | 0.751 | 0.609 |
| 5 | 0.904 | 0.265 | 0.752 | 0.595 |
| 6 | 0.906 | 0.260 | 0.757 | 0.616 |
| 7 | 0.908 | 0.252 | 0.756 | 0.591 |
| 8 | 0.911 | 0.249 | 0.757 | 0.629 |
| 9 | 0.912 | 0.244 | 0.755 | 0.635 |
| 10 | 0.914 | 0.240 | 0.755 | 0.637 |
| 11 | 0.915 | 0.240 | 0.757 | 0.626 |
| 12 | 0.916 | 0.235 | 0.754 | 0.644 |
| 13 | 0.917 | 0.231 | 0.758 | 0.653 |
| 14 | 0.917 | 0.234 | 0.756 | 0.650 |
| 15 | 0.918 | 0.231 | 0.759 | 0.654 |
| 16 | 0.920 | 0.227 | 0.759 | 0.657 |
| 17 | 0.920 | 0.229 | 0.760 | 0.631 |
| 18 | 0.920 | 0.227 | 0.759 | 0.646 |
| 19 | 0.921 | 0.228 | 0.759 | 0.645 |

c

**
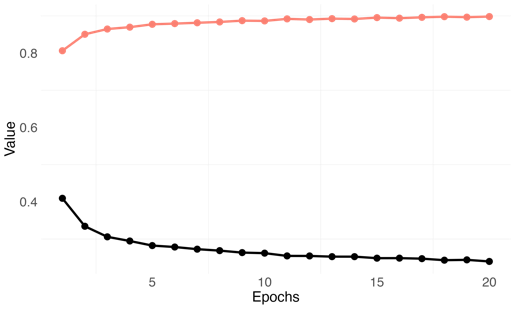
**

**a** Five-fold cross validation curves for cSCCNet Model 2 on the training cohort (n = 172) after 20 epochs, with training accuracy in red and validation accuracy in blue. The mean k-fold (bottom right curve) achieved tile-level accuracies of accuracies of 0.92 for training and 0.76 for validation after 20 epochs. **b** Mean k-fold results for cSCCNet Model 2 on the training cohort (bottom right curve in Supp Fig 2a) for 20 epochs. **c** Final model training curve for Model 2 (retrained on the entire training cohort, n = 172), with training accuracy in red and training loss in black. The model reached accuracy 0.90 and loss 0.24 after 20 epochs.

**Supplementary Figure 3. Model 2 threshold selection**


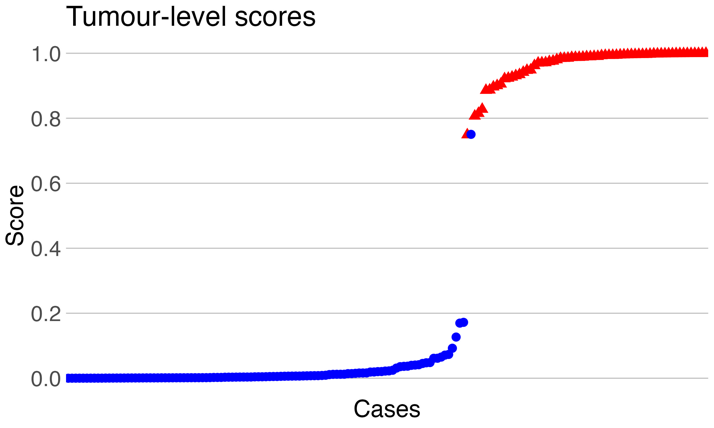
**
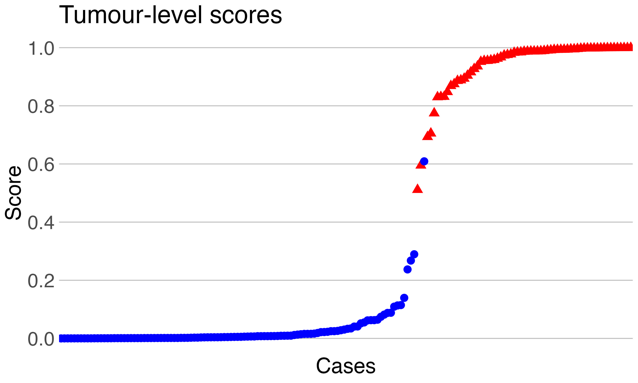

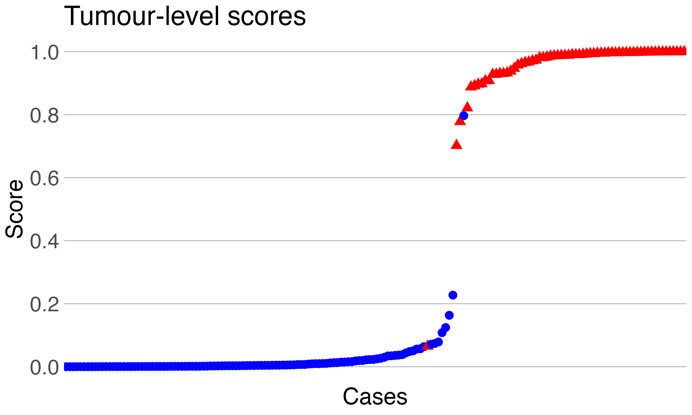
**

c

a

b

d

| **Threshold** | **TP** | **FP** | **FN** | **TN** | **accuracy** | **sensitivity** | **specificity** | **PPV** | **NPV** |
| --- | --- | --- | --- | --- | --- | --- | --- | --- | --- |
| >50% ≥ 0.5 | 63 | 1 | 1 | 107 | 99% | 98% | 99% | 98% | 99% |
| >30% ≥ 0.7 | 63 | 2 | 1 | 106 | 98% | 98% | 98% | 97% | 99% |
| >20% ≥ 0.7 | 64 | 4 | 0 | 104 | 98% | 100% | 96% | 94% | 100% |
| >10% ≥ 0.9 | 64 | 2 | 0 | 106 | 99% | 100% | 98% | 97% | 100% |
| Median >0.8 | 61 | 0 | 3 | 108 | 98% | 95% | 100% | 100% | 97% |
| Median >0.2 | 63 | 2 | 1 | 106 | 98% | 98% | 98% | 97% | 99% |

e

| **Threshold** | **TP** | **FP** | **FN** | **TN** | **accuracy** | **sensitivity** | **specificity** | **PPV** | **NPV** |
| --- | --- | --- | --- | --- | --- | --- | --- | --- | --- |
| >50% ≥ 0.5 | 11 | 1 | 3 | 25 | 90% | 79% | 96% | 92% | 89% |
| >30% ≥ 0.7 | 13 | 2 | 1 | 24 | 93% | 93% | 92% | 87% | 96% |
| >20% ≥ 0.7 | 14 | 4 | 1 | 21 | 88% | 93% | 84% | 78% | 95% |
| >10% ≥ 0.9 | 13 | 3 | 2 | 22 | 88% | 87% | 88% | 81% | 92% |
| Median >0.8 | 10 | 0 | 4 | 26 | 90% | 71% | 100% | 100% | 87% |
| Median >0.2 | 13 | 1 | 1 | 25 | 95% | 93% | 96% | 93% | 96% |

**a** Scatterplot showing Model 2 median tile scores for metastasising cases as red triangles and non-metastasising cases as blue circle, including. All tiles within the pathologist-annotated regions of interest (ROI) were included for all cases in the training cohort (n=172). **b** To improve the separation between low- and high-risk tumours, borderline tiles (with scores 0.3-0.7) were excluded. Scatterplot showing Model 2 median tile scores, for tiles within pathologist ROI and after excluding tiles with borderline scores. A median score >0.2 was selected as the definition for ‘high-risk’ cSCC, based on graphical representations and accuracy statistics. **c** Both models were then used in series in the training cohort (n=172). Scatterplot showing Model 2 median tile scores, for tiles selected by Model 1 and after excluding borderline tiles. **d** Data from the training cohort (n=172) show comparison of various Model 2 thresholds in predicting risk of cSCC metastasis when both models were used in series and after excluding borderline tiles. Thresholds shown: ‘>50% ≥ 0.5’ (>50% of tiles have score ≥0.5), ‘>30% ≥ 0.7’ (>30% of tiles have score ≥ 0.7), ‘>20% ≥ 0.7’ (>20% of tiles have score ≥ 0.7), ‘>10% ≥ 0.9’ (>10% of tiles have score ≥ 0.9), median tile score > 0.8 and median tile score > 0.2. **e** Data from the testing cohort (n=40) validating the same Model 2 thresholds, when both models were used in series and after excluding borderline tiles. FN: false negatives; FP: false positives; NPV: negative predictive value; PPV: positive predictive value; TN: true negatives; TP: true positives.

**Supplementary Figure 4.** **Model 2 evaluation**

a


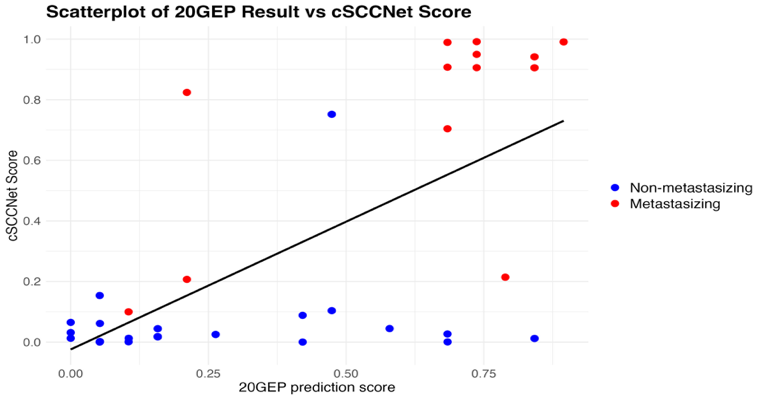


r= 0.66

b

**
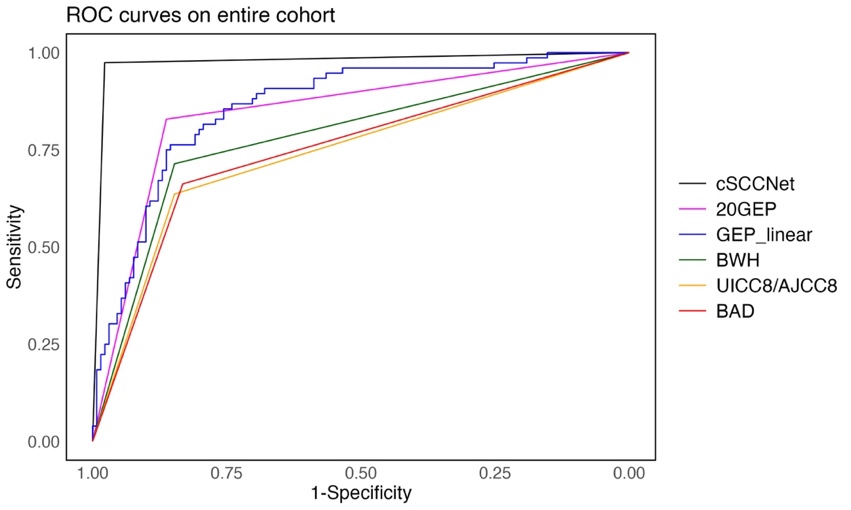
**

d

**
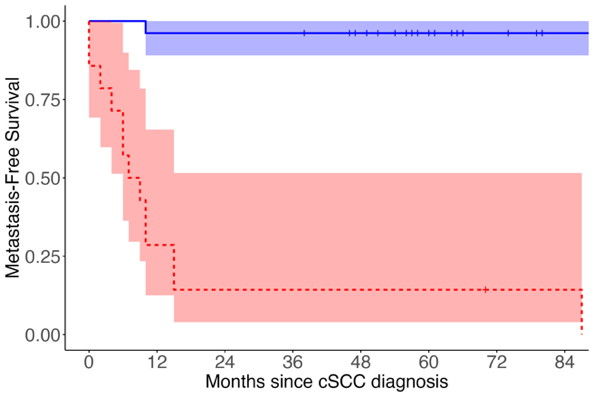
**


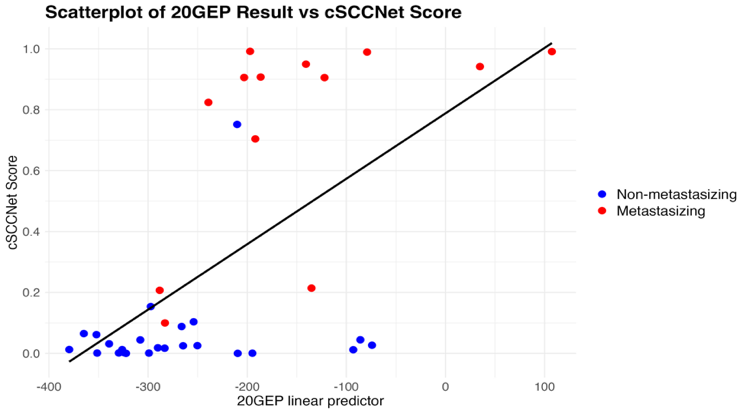


c

r= 0.61


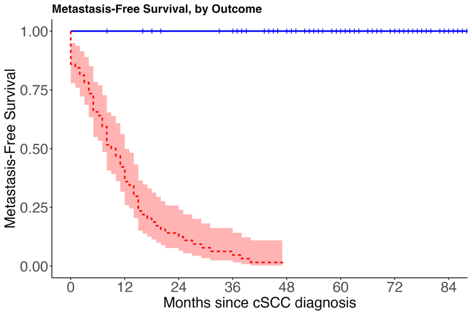


g

f

e


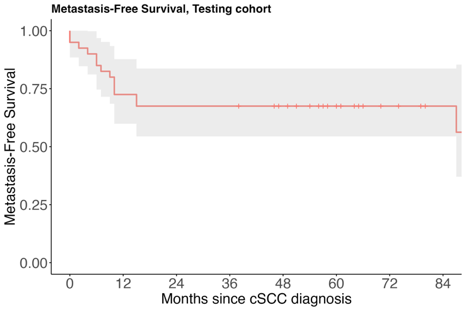

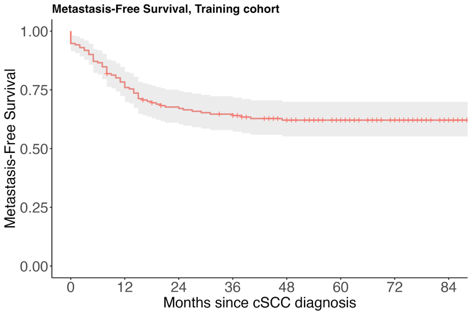


i

h

| **Variable** | **Hazard Ratio** | **95% CI** | **p-value** |
| --- | --- | --- | --- |
| cSCCNet | 53.4 | 6.8-417.1 | 1e-4 |
| 20-GEP | 7.3 | 2.0-27.1 | 0.003 |
| UICC8/AJCC8 | 4.4 | 1.5-12.9 | 0.006 |
| BWH | 4.7 | 1.5-14.5 | 0.008 |
| BAD | 4.4 | 1.5-12.9 | 0.006 |
| Diameter | 1.1 | 1.03-1.1 | 1.4e-5 |
| Differentiation | 14.4 | 1.9-110 | 0.01 |
| Thickness | 1.13 | 1.0-1.3 | 0.04 |
| LVI | 6.0 | 1.6-22.9 | 0.009 |


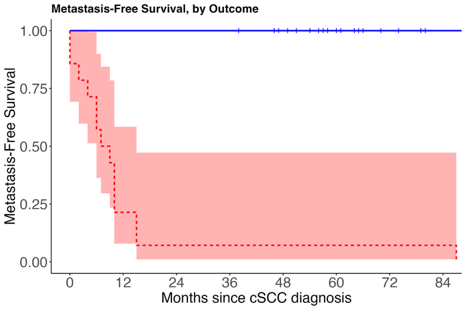


k

j

| **Variable** | **Hazard**  **Ratio** | **95% CI** | **p-value** |
| --- | --- | --- | --- |
| Age | 0.94 | 0.88-1 | 0.05 |
| Sex | 2.9 | 0.3-29.3 | 0.37 |
| Differentiation | 0.6 | 0.05-8.3 | 0.74 |
| UICC8/AJCC8 | 2.8 | 0.8-9.5 | 0.10 |
| cSCCNet | 74.9 | 4.8-1170.6 | 0.002 |

| **Variable** | **Hazard**  **Ratio** | **95% CI** | **p-value** |
| --- | --- | --- | --- |
| Age | 0.93 | 0.88-0.99 | 0.02 |
| Sex | 2.2 | 0.2-21.7 | 0.49 |
| BWH | 2.2 | 0.7-7.4 | 0.20 |
| cSCCNet | 68.4 | 6.0-785.3 | 6.9e-4 |

**a** Receiver operating characteristic (ROC) curves are shown for cSCCNet (black), the 20-gene expression profile (20-GEP) prediction score based on k-nearest neighbours analysis (20GEP, purple) and linear predictor (GEP_linear, blue), Brigham and Women’s Hospital classification (BWH, green), 8th edition Union for International Cancer Control classification/8th edition American Joint Committee on Cancer staging (UICC8/AJCC8, yellow), and British Association of Dermatologists’ cSCC guidelines (BAD, red) on the entire cohort (n = 212). This includes the training cases for the cSCCNet and 20-GEP models, which might bias their performance. The area under the ROC curves (AUC) and their 95% confidence intervals were: 0.98 (0.95-1) for cSCCNet, 0.85 (0.79-0.90) for 20-GEP prediction score, 0.86 (0.81-0.91) for the 20-GEP linear predictor, 0.74 (0.68-0.80) for UICC8/AJCC8, 0.78 (0.72-0.84) BWH, and 0.74 (0.69-0.81) for BAD. For 37 cases in the testing cohort where complete data were available, the Pearson correlation between the cSCCNet score and 20-GEP test showed a moderate positive correlation: **b** 0.66 (p = 9e-6) for the 20-GEP prediction score and **c** 0.61 (p = 7e-5) for the 20-GEP linear predictor score. Kaplan-Meier curves showing metastasis-free survival after cSCC diagnosis for: **d** the entire cohort (n=212) stratified by cSCCNet prediction, with high-risk cases in red and low-risk cases in blue, **e** the training cohort (n=172), **f** the testing cohort (n=40), **g** the training cohort, stratified by cSCCNet prediction, and **h** the testing cohort, stratified by cSCCNet prediction. **i** On univariate analysis, features predictive of metastasis (Wald test, p <0.05) in the testing cohort included the cSCCNet classification, 20-GEP, UICC8/AJCC8, BWH, BAD Very High-risk grade, tumour diameter, differentiation, thickness, and presence of lymphovascular invasion (LVI). Age, sex, site of primary cSCC and presence of perineural invasion were not statistically significant in the testing cohort; however, all were significant (p <0.05) when assessed in the entire cohort (n=212), suggesting an impact of sample size. Margin status was not a significant predictor of outcome. **j** On multivariate analysis for predicting the risk of metastasis in the testing cohort (n=35), cSCCNet was a significant predictor, independently of age, sex, tumour differentiation, or UICC8/AJCC8. **k** cSCCNet was also a significant predictor when multivariate analysis was repeated with BWH. As differentiation is already included within BWH, it was not included as a separate variable. 20-GEP: 20-gene expression profile; AJCC8: 8^th^ edition American Joint Committee on Cancer staging; BAD: British Association of Dermatologists’ cSCC guidelines; BWH: Brigham and Women’s Hospital classification; CI: confidence interval; cSCC: cutaneous squamous cell carcinoma; UICC8: 8th edition Union for International Cancer Control classification.

**Supplementary Figure 5. Evaluation of model training strategy using centre-split cross-validation**

a

| **Model** | **Training cohort** | **Testing cohort** |
| --- | --- | --- |
| cSCCNet | 108 (63%) non-metastasizing  64 (37%) metastasizing | 26 (65%) non-metastasizing  14 (35%) metastasizing |
| Model BCD | 113 (73%) non-metastasizing  42 (27%) metastasizing | Centre A:  21 (49%) non-metastasizing  22 (51%) metastasizing |
| Model ABD | 103 (80%) non-metastasizing  25 (20%) metastasizing | Centre C:  31 (44%) non-metastasizing  39 (56%) metastasizing |

c

b

a


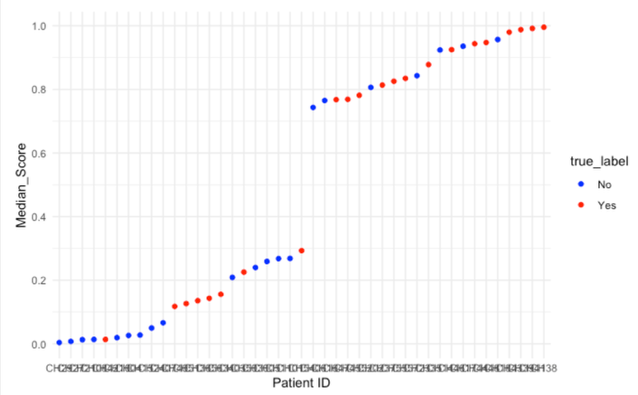

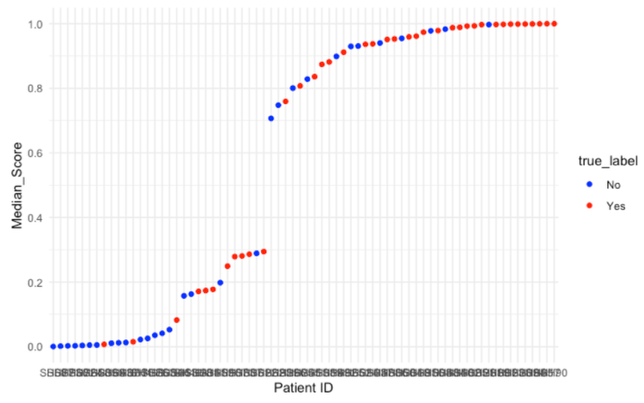


d

|  | **AUC**  **(95% CI)** | **Accuracy** | **Specificity** | **Sensitivity** | **NPV** | **PPV** | **TN** | **TP** | **FN** | **FP** |
| --- | --- | --- | --- | --- | --- | --- | --- | --- | --- | --- |
| **cSCCNet** | 0.95  (0.87-1) | 95% | 96% | 93% | 96% | 93% | 25 | 13 | 1 | 1 |
| **Model BCD** | 0.58  (0.43-0.72) | 58% | 43% | 73% | 60% | 57% | 9 | 16 | 6 | 12 |
| **Model ABD** | 0.71  (0.61-0.82) | 73% | 58% | 85% | 75% | 72% | 18 | 33 | 6 | 13 |

**a** Table showing the number of cases contributing to the training and testing cohorts for each model. cSCCNet was trained and tested on cases from all four centres; the 80:20 training:testing split was random and stratified by tumour outcome and contributing centre. Model BCD was trained on all cases from centres B,C and D, and tested on all cases from centre A. Model ABD was trained on all cases from centres A,B and D, and tested on all cases from centre C. Scatterplots showing **b** Model BCD median tile scores for the Testing cohort (centre A), and **c** Model ABD median tile scores for the Testing cohort (centre C), for tiles within pathologist ROI and after excluding borderline tiles (with scores 0.3-0.7). **d** Table showing performance in predicting risk of cSCC metastasis, based on slide-level predictions of cSCCNet, Model BCD, and Model ABD. 95% CI: 95% confidence interval. AUC: area under the receiver operating characteristic curve; FN: false negatives; FP: false positives; NPV: negative predictive value; PPV: positive predictive value; TN: true negatives; TP: true positives.

**Supplementary Figure 6. Review of incorrect cases**


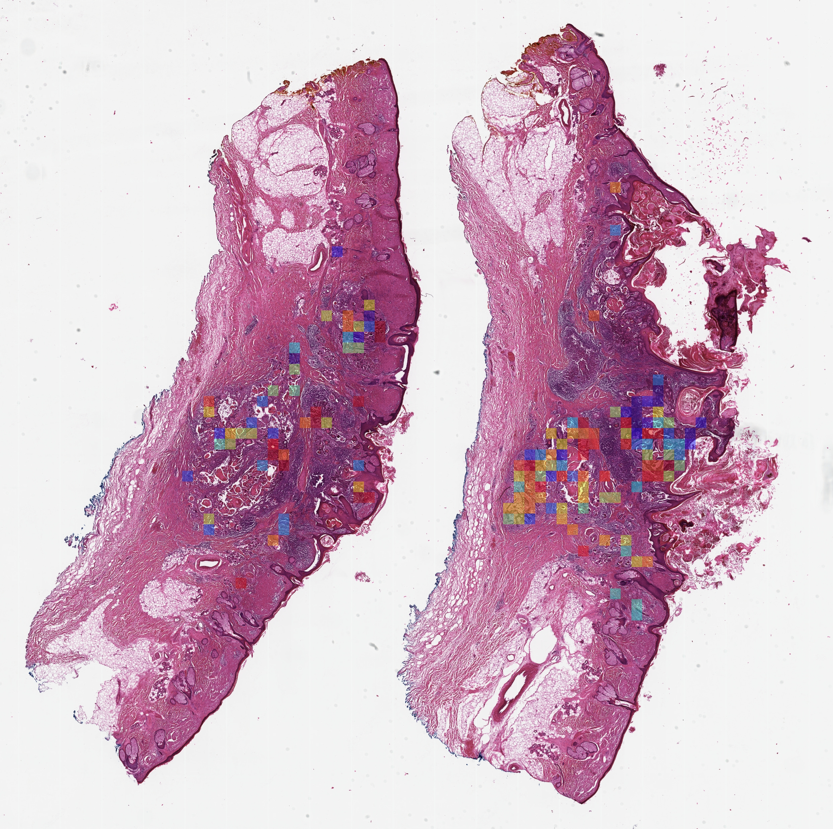

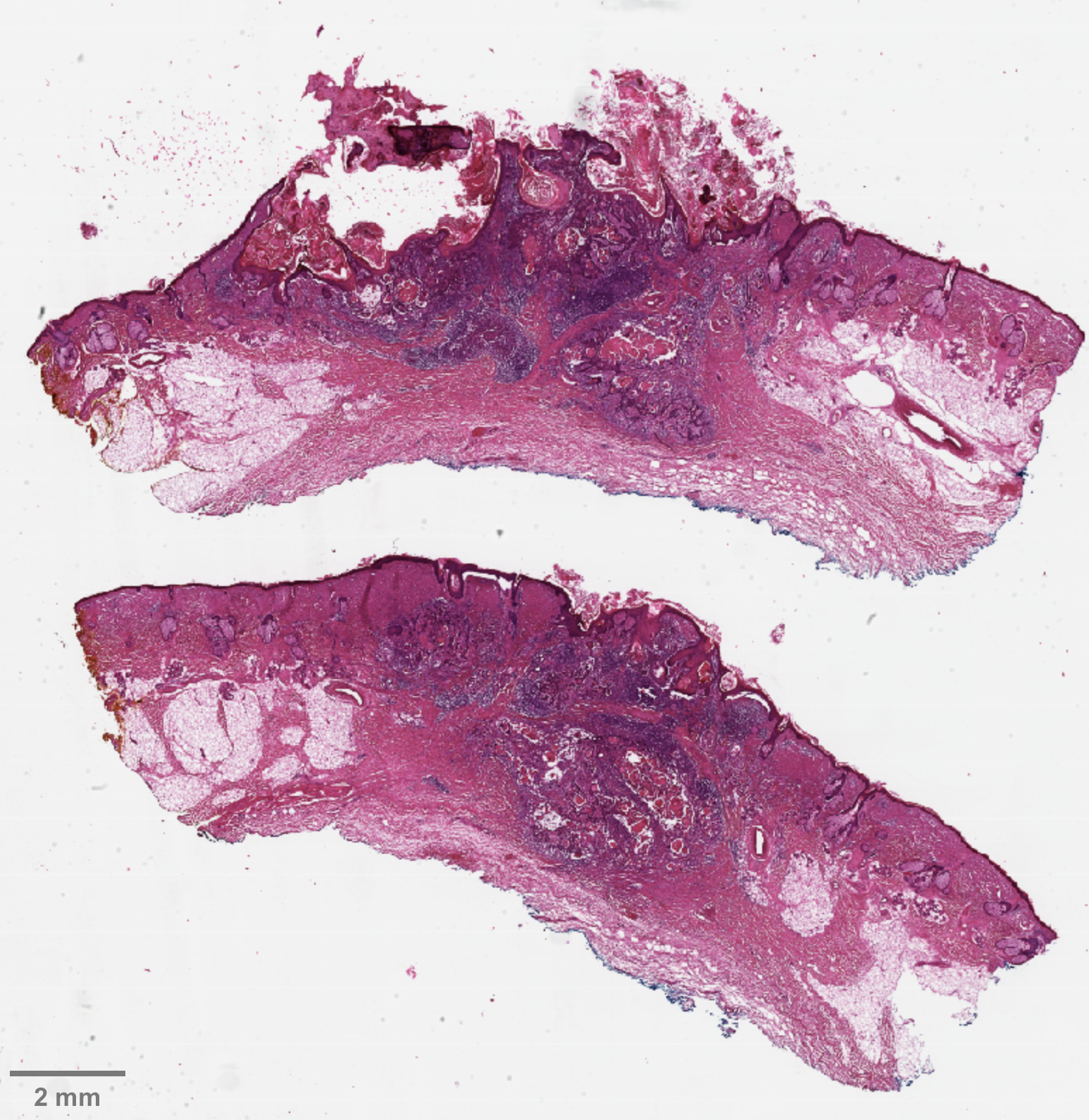


a

High risk (0.75)


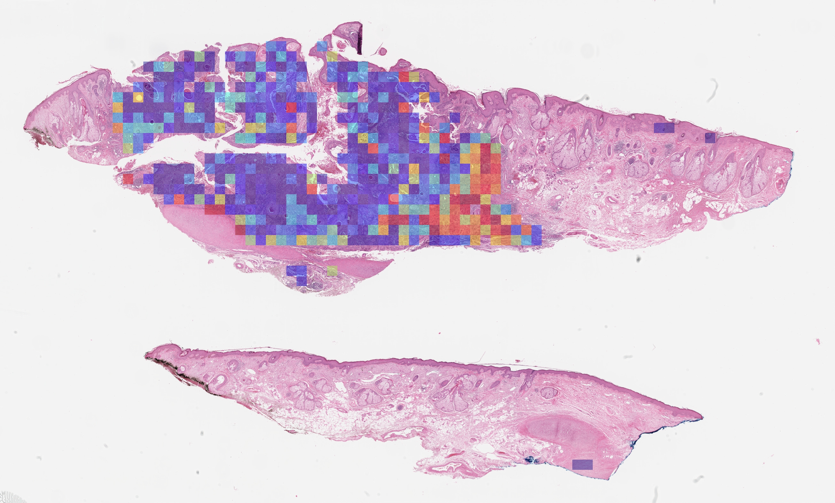

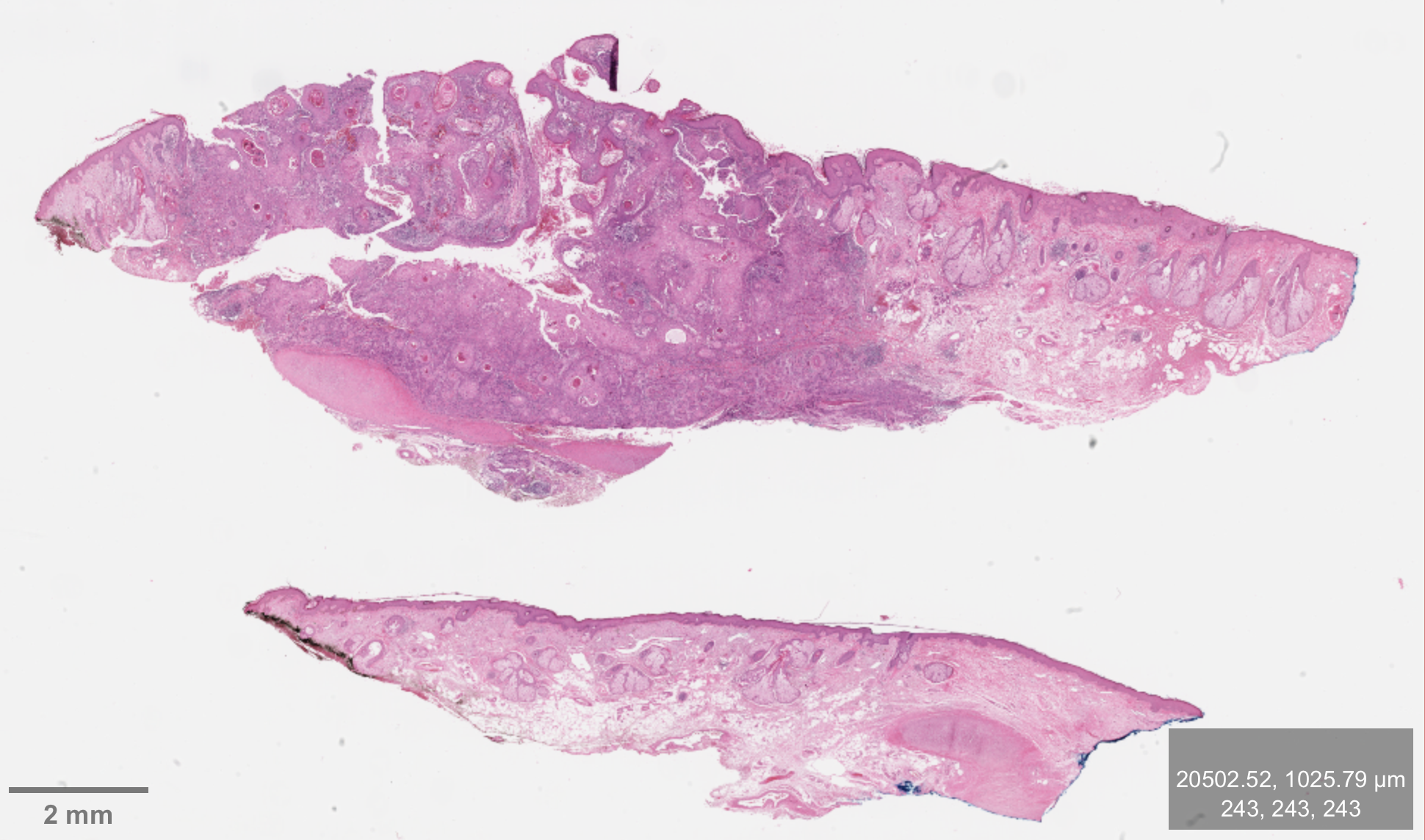


bb

Low risk (0.10)

Heatmaps for the testing cohort cases misclassified by cSCCNet (n=2/40), with Model 2 tile scores converted to colour using a blue to red scale, for scores 0-1 (low to high-risk). The tumour-level aggregate scores are on the top right corner of each case, with scores >0.20 representing ‘high-risk’ tumours. **a** Non-metastasising scalp cSCC classified as high-risk by the cSCCNet model and by staging criteria (UICC8/AJCC8 T3 and BWH T2b). It is poorly differentiated and invades beyond subcutis. Heatmaps show that Model 1 had failed to select >60% of the ROI; the small number of tiles passed to Model 1 contained poorly differentiated carcinoma. **b** A metastasising pinna cSCC with incomplete excision margins was classified as low-risk by the cSCCNet model. The majority of the tumour was moderately-differentiated with good keratinisation; however, there was extension beyond cartilage. It was high grade on staging criteria (UICC8/AJCC8 T3 and BWH T2b). A small area of poorly differentiated carcinoma was present and was classified as ‘high-risk’ by the model. AJCC8: 8^th^ edition American Joint Committee on Cancer staging; BAD: British Association of Dermatologists’ cSCC guidelines; BWH: Brigham and Women’s Hospital classification; cSCC: cutaneous squamous cell carcinoma; ROI: region of interest; UICC8: 8th edition Union for International Cancer Control classification.

**Supplementary Table 1. Baseline clinicopathological characteristics**

|  | **Training cohort**  **n = 172**^a^ | **Testing cohort**  **n=40** |
| --- | --- | --- |
| Metastasising cases  Non-metastasising cases | 64 (37)  108 (63) | 14 (35)  26 (65) |
| Contributing centres | A: 37  B: 14  C: 56  D: 65 | A: 6  B: 3  C: 14  D: 17 |
| Age, years | 80 (71-84) | 82 (75-86) |
| **Sex**  Male  Female | 115 (67)  57 (33) | 29 (73)  11 (28) |
| **Site**  Head and Neck | 112 (65) | 24 (60) |
| **Tumour diameter**  Median, mm  >=20 mm | 15 (10-23)  58/167 (35) | 15 (10-25)  15 (38) |
| **Differentiation**  Poorly differentiated  Moderately differentiated  Well differentiated | 87 (51)  63 (37)  22 (13) | 21 (53)  13 (33)  6 (15) |
| **Thickness, mm**  Median  >6mm | 3  24/170 (14) | 3  3/36 (8) |
| **Invasion to**  Dermis  Subcutis  Beyond subcutaneous fat | 89 (53)  45 (27)  35 (21) | 24 (60)  9 (23)  7 (18) |
| Perineural invasion | 22/170 (13) | 3/39 (8) |
| Lymphovascular invasion | 11/167 (7) | 3/38 (8) |
| **Follow-up, months**^b^ | 53 (14-74) | 55 (10-76) |
| **Time to metastasis, months^c^** | 10 (4-15), range 0-47 | 8 (5-10), range 0-87 |
| **UICC8/AJCC8**  pT1  pT2  pT3 | 96 (57)  16 (9)  57 (34) | 22 (56)  5 (13)  12 (31) |
| **BWH**  T1  T2a  T2b  T3 | 61 (36)  45 (27)  58 (34)  5 (3) | 12 (31)  15 (38)  10 (26)  2 (5) |
| **BAD**  LR  HR  VHR | 45 (27)  64 (38)  60 (36) | 11 (28)  15 (38)  13 (33) |

This table does not include the 15 cases used to train Model 1 only. Numbers in brackets are percentages or interquartile range, as appropriate. There were no statistically significant differences (p > 0.05) between the training and testing cohorts, using the Mann-Whitney U test for continuous variables and Fisher's exact test for categorical variables. Although all cSCC were treated with wide local excision, 30/210 tumours (21/172 training and 9/40 testing) had incomplete excision margins, and all had either re-excision or adjuvant radiotherapy, except one case in the training cohort which had already metastasized at the time of presentation of the primary lesion. ^a^172 tumours from 171 patients. ^b^Follow-up was censored at metastasis, death, or loss to follow-up, whichever occurred first. ^c^Results for patients with metastasizing cSCC (n=78). AJCC8: American Joint Committee on Cancer staging manual; BAD: British Association of Dermatologists’ cSCC guidelines; BWH: Brigham and Women’s Hospital classification; HR: high risk; LR: low risk; UICC8: the 8th edition Union for International Cancer Control classification; VHR: very high risk.
